# Glycomacropeptide as an Efficient Agent to Fight Pathophysiological Mechanisms of Metabolic Syndrome

**DOI:** 10.3390/nu16060871

**Published:** 2024-03-17

**Authors:** Mathilde Foisy Sauvé, Francis Feldman, Alain Théophile Sané, Mireille Koudoufio, Natalie Patey, Schohraya Spahis, James Butcher, Haonan Duan, Daniel Figeys, Valérie Marcil, Alain Stintzi, Emile Levy

**Affiliations:** 1 Research Centre, Sainte-Justine University Health Center, Montreal, QC H3T 1C5, Canada; mathilde.foisy.sauve@umontreal.ca (M.F.S.); francis.feldman@umontreal.ca (F.F.); sanealaintheo@gmail.com (A.T.S.); mireille.koudoufio@umontreal.ca (M.K.); natalie.patey.med@ssss.gouv.qc.ca (N.P.); schohraya.spahis.hsj@ssss.gouv.qc.ca (S.S.); valerie.marcil@umontreal.ca (V.M.); 2 Department of Nutrition, Université de Montréal, Montreal, QC H3T 1A8, Canada; 3 Department of Pathology and Cell Biology, Université de Montréal, Montreal, QC H3T 1J4, Canada; 4 Department of Biochemistry & Molecular Medicine, Université de Montréal, Montreal, QC H3T 1J4, Canada; 5 Department of Biochemistry, Microbiology, and Immunology, Ottawa Institute of Systems Biology, University of Ottawa, Ottawa, ON K1H 8M5, Canada; jbutcher@uottawa.ca (J.B.); hduan084@uottawa.ca (H.D.); dfigeys@uottawa.ca (D.F.); astintzi@uottawa.ca (A.S.); 6 School of Pharmaceutical Sciences, University of Ottawa, Ottawa, ON K1H 8M5, Canada

**Keywords:** milk peptide, metabolic syndrome, gut–liver axis, inflammation, oxidative stress, endoplasmic reticulum stress, fatty acids

## Abstract

There is currently a growing interest in the use of nutraceuticals as a means of preventing the development of complex diseases. Given the considerable health potential of milk-derived peptides, the aim of this study was to investigate the protective effects of glycomacropeptide (GMP) on metabolic syndrome. Particular emphasis was placed on the potential mechanisms mitigating cardiometabolic disorders in high-fat, high-fructose diet-fed mice in the presence of GMP or Bipro, an isocaloric control. The administration of GMP for 12 weeks reduced obesity, hyperglycemia and hyperinsulinemia caused by a high-fat, high-fructose diet, resulting in a decline in insulin resistance. GMP also lessened systemic inflammation, as indicated by decreased circulating inflammatory cytokines. In the intestinal and hepatic tissues, GMP improved homeostasis by increasing insulin sensitivity and attenuating high-fat, high-fructose-induced inflammation, oxidative stress and endoplasmic reticulum stress. Biochemical and histological analyses revealed improved hepatic steatosis and fatty acid composition in the livers of high-fat, high-fructose diet-fed mice treated with GMP compared to Bipro. A trend toward a decrease in bile acids without any marked changes in intestinal microbiota composition characterized GMP-treated animals compared to those administered Bipro. GMP offers considerable potential for fighting metabolic syndrome-related components and complications given its beneficial effects on risk factors such as inflammation, oxidative stress and endoplasmic reticulum stress without involving the intestinal microbiota.

## 1. Introduction

Metabolic syndrome (MetS), a widespread condition, consists of several physiological disorders, including central obesity, reduced high-density lipoproteins, elevated triglycerides (TGs), high blood pressure and raised fasting blood glucose [1,2]. Each component, but especially the synergetic effect of their sum, contributes to the development of serious complications such as type 2 diabetes, non-alcoholic fatty liver disease (NAFLD) and atherosclerosis [3,4]. Although excessive energy intake and the Western dietary pattern exert a major impact on MetS progression, its precise etiology remains nebulous. 

First-line therapy for MetS includes lifestyle modifications such as diet modification and physical activity; however, these measures are often insufficient to normalize risk factors, leading to the use of pharmaceutical agents. As MetS represents a cluster of multiple risk factors, it requires polypharmacological treatment such as angiotensin-converting enzyme inhibitors or diuretics for lowering high blood pressure, fibrates for controlling abnormally increased TG levels, and peroxisome proliferator-activated receptor-γ agonists, insulin-sensitizing agents, glucagon-like peptide-1 receptor agonists, dipeptidyl peptidase-4 inhibitors or sodium-glucose cotransporter-2 inhibitors for reducing hyperglycemia [5]. However, there is no single pharmaceutical therapy that can effectively address all underlying causes simultaneously [3]. Given the side effects of these various drugs and the interferences between them, there is an urgent need to adopt a comprehensive and valuable approach that can simultaneously treat all aspects of MetS.

Non-pharmaceutical alternatives, including nutraceuticals and phytotherapy, are currently attracting growing interest as a means of hindering the development of complex diseases or treating them safely. Nutraceuticals can communicate with diverse cellular compartments, transcription factors, regulatory genes and metabolic pathways [6,7]. In this context, human and bovine milk contain a myriad of bioactive peptides, which provide remarkable health benefits [8,9,10]. For example, many of these milk constituents are endowed with advantageous properties and physiological functions, resulting in tangible benefits such as calcium absorption, bone health, improved immunity and protection against infections [11].

Recently, our laboratory highlighted the ability of glycomacropeptide (GMP) to counteract oxidative stress (OxS) and inflammation in intestinal Caco-2/15 cells [12], along with the amelioration of some aspects of MetS, such as insulin resistance (IR). In a subsequent study, we validated these findings using a murine model exposed to a high-fat, high-sucrose diet [13]. Interestingly, the administration of GMP to high-fat, high-sucrose diet-fed mice resulted in significant attenuation of IR, which is central to MetS pathogenesis. Furthermore, GMP displayed powerful antioxidant and anti-inflammatory properties in the liver, besides attenuating lipid accumulation, suggesting that GMP could be effective for NAFLD as well [13]. 

Several studies have reported links between MetS and the gut microbiota [14,15,16,17]. Notably, it has been suggested that disturbances in the composition and diversity of gut microbes may contribute to MetS development [18,19]. In addition, the gut microbiota is thought to be involved in the regulation of energy metabolism through the production of short-chain fatty acids (SCFA), which can modulate glucose and lipid metabolism [20,21,22]. Given that GMP can be absorbed without digestive degradation and has been suggested to alter the gastrointestinal microbiota [23,24,25,26], we thought it would be of great interest to examine the regulatory role of GMP in the composition, diversity and functional potential of the gut microbiota while focusing on MetS components in mice fed a high-fat, high-fructose (HFHF) diet. As the gut–liver axis represents a key player in the progression of MetS complications, we investigated which mechanisms including inflammation, OxS and endoplasmic reticulum (ER) stress were potentially triggered by GMP in the gut and the liver.

## 2. Materials and Methods

### 2.1. Glycomacropeptide

GMP (as the bioactive protein) and Bipro were obtained from Agropur Dairy Cooperative (Eden Prairie, MN, USA). Importantly, Bipro was selected as an isocaloric control since it contains the same amino acids as GMP, but in a random order. 

### 2.2. Animals

Eight-week-old C57BL/6 male mice (*n* = 36) were purchased from Charles River and maintained in a temperature- and humidity-controlled environment (22 ± 1 °C) on a 12 h daylight cycle with free access to food and water. During the first week, mice were acclimatized with a chow diet from Harlan Laboratories (18.6% protein, 44.2% carbohydrates and 6.2% fat; 3.1 kcal/g). To determine the effect of GMP on diet-induced MetS, mice were then separated into individual cages and randomly assigned to three different dietary conditions for 12 weeks. Mice were fed either a standard chow or a high-fat, high-fructose diet (HFHF). Chow-fed animals (*n* = 12) were administered daily a water vehicle while HFHF diet-fed animals received oral doses of Bipro, an isocaloric control (HFHF + Bipro; *n* = 12), or glycomacropeptide (HFHF + GMP; *n* = 12) (200 mg/kg body weight). The HFHF diet consisted of a high-fat, high-sucrose diet from Research Diets (15% protein, 20% sucrose and 65% fat; 5.2 kcal/g) in addition to 30% fructose in drinking water (Supplementary data, Figure S1).

Body weight and food intake were measured twice a week. Stool samples were freshly collected at baseline and at weeks 6, 9 and 11 and immediately stored at −80 °C for subsequent metagenomics sequencing and SCFA analysis. After 12 weeks of treatment, animals were anesthetized in chambers (saturated with isoflurane) and euthanized by cardiac puncture. Blood was collected in EDTA-coated tubes and plasma was separated from cells by centrifugation at 3000× *g* for 20 min at 4 °C. Organs such as small intestinal segments, liver and adipose tissues were dissected, weighed and flash-frozen in liquid nitrogen before being stored at −80 °C for further experiments. Some tissue samples were placed in TRIzol for mRNA determinations, placed in radioimmunoprecipitation assay (RIPA) buffer for protein analyses or fixed in 10% neutral buffered formalin for histological examination. All animal manipulations were approved by the Institutional Animal Care Committee of the Sainte-Justine UHC Research Center.

### 2.3. Glucose and Lipid Homeostasis

Blood was collected from the tail at weeks 0, 6 and 10 after an overnight fast (12 h), and glycemia was measured with an Accu-Check glucometer. Plasma was then separated as described above and insulin, TG, and total cholesterol (TC) concentrations were assessed using commercial kits (Millipore, Billerica, MA, USA & Randox Laboratories, Crumlin, UK). The Homeostatic Model Assessment for Insulin Resistance (HOMA-IR) index was then calculated using the following formula: fasting insulinemia (mUI/mL) × fasting glycemia (mM)/22.5. Plasma bile acids were assessed using liquid chromatography coupled with tandem mass spectrometry, as previously described [27].

*Lipid extraction in tissue.* Approximately 0.1 g of liver tissue was homogenized in 1 mL of PBS-EDTA buffer and lipids were extracted in a 2:1 chloroform/methanol solution overnight at 4 °C. After evaporation of the lower phase, lipids were resuspended in 400 μL of H_2_O, and TG and TC concentrations were determined as described above. Phospholipids were measured using the Bartlett method [28].

### 2.4. Analytical Methods

At sacrifice, plasma was collected to measure high molecular weight (HMW) adiponectin concentrations with the Mouse HMW & Total Adiponectin Elisa kit (ALPCO, Salem, NH, USA). Plasma samples (*n* = 8/group) were also assessed for inflammatory factors by a 32-multiplex cytokine array (Eve technologies, Calgary, AB, Canada).

### 2.5. Histological Analyses

As previously mentioned, at sacrifice, colon, liver and mesenteric adipose tissue samples were fixed in 10% neutral buffered formalin, dehydrated in gradient ethanol washing series and embedded in paraffin. For histological evaluation, 3 μm thick tissue sections were stained with either hematoxylin-phloxine saffron (HPS) or hematoxylin-eosin (HES) and examined under an optic microscope by a pathologist who was blinded to the experimental protocol. Images of stained tissues were captured by Zeiss Imager A1. Measurements were taken with the AxioVision software (https://www.micro-shop.zeiss.com/en/us/system/software+axiovision-axiovision+program-axiovision+software/10221/#variants, accessed on 23 September 2021) for colon and liver tissues (*n* = 4), as previously described [13,29]. 

*Automated analysis of histological images using Adiposoft.* The protocol from Galarraga was followed to determine the adipocyte numbers and cell area [30]. Briefly, representative histological images of mice mesenteric adipose tissues (*n* = 4) were analyzed using ImageJ version 1.53a (https://imagej.nih.gov/ij/, accessed on 23 September 2021) with the Adiposoft version 1.16 plug-in (https://imagej.net/plugins/adiposoft, accessed on 23 September 2021). As with the Choi procedure [31], (i) the scale was set at 1.02 micron/pixel, (ii) adipocytes with a diameter < 30 μm or >300 μm were removed and (iii) adipocytes on the edges were excluded. After automated analysis, images were reviewed and manually edited if needed. To minimize data distortion caused by artifacts, we excluded values less than 500 μm^2^ and greater than 15,000 μm^2^ [32]. 

### 2.6. RNA Isolation, Reverse Transcription and Quantitative PCR Analyses

Intestinal and hepatic tissue samples were homogenized in TRIzol reagent and total RNA was extracted. RNA concentration and purity were determined by the 260/280 ratio using a Biodrop Touch Duo spectrophotometer (Montreal Biotech Inc., Dorval, QC, Canada). Agarose gel electrophoresis was also performed to assess RNA integrity. Complementary DNA was obtained by reverse transcribing 1 μg of total RNA with Superscript VILO Master Mix (Invitrogen, Waltham, MA, USA). Gene expression was analyzed by quantitative RT-PCR (qRT-PCR) using the 7500 Fast Real-Time PCR System (Applied Biosystems, Waltham, MA, USA) with PowerUp SYBR Green Master Mix (Applied Biosystems, Waltham, MA, USA). The thermal profile included an initial denaturation at 95 °C for 30 s, followed by 40 cycles of denaturation at 95 °C for 3 s, and a combined annealing/extension step at 60 °C for 30 s. Expression levels of target-gene mRNAs were calculated by the 2^−ΔΔCT^ method. mRNA expression of each gene was normalized to β-actin as a reference gene. The list of all primers can be found in Supplementary Table S1. 

### 2.7. Western Blot Analysis

*Total homogenates.* Liver and colon tissue samples were homogenized in cold RIPA buffer supplemented with a cocktail of protease and phosphatase inhibitors. Supernatant proteins were obtained after centrifugation at 3000× *g* for 10 min at 4 °C, and protein concentration was determined using the Bradford assay (Bio-Rad, Hercules, CA, USA). Samples (10 µg) were then denatured at 95 °C for 5 min in a sample buffer containing sodium dodecyl sulphate (SDS) and β-mercaptoethanol, separated on a 10% sodium dodecyl sulphate polyacrylamide gel and electroblotted onto nitrocellulose membranes. Nonspecific binding sites of the membranes were blocked with Tris-buffered saline [20 mM Tris–HCl (pH 7.5), 137 mM NaCl] containing 0.1% Tween 20 and 5% non-fat dry milk for 60 min at room temperature. Membranes were then incubated overnight at 4 °C in a blocking solution with primary antibodies (Supplementary Data, Table S2). The relative amount of primary antibody was detected with a species-specific horseradish peroxidase-conjugated secondary antibody using the ChemiDoc MP Imaging System (Bio-Rad, Hercules, CA, USA). All data were expressed as the ratio of target protein to β-actin, GAPDH or lamin, which served as housekeeping proteins. Of note, Western blots are most accurate when both forms of the protein of interest (total and phosphorylated) are applied to the same blot and, therefore, share the same housekeeping gene. However, to avoid stripping and re-probing the same membrane, which can reduce resolution, we analyzed the total and phosphorylated forms of PERK, AKT, p38 and SAPK/JNK using different blots. Each form was thus evaluated against its housekeeping protein before dividing the results of the phosphorylated form by the total form.

*Cytosol and nucleus isolation.* Liver and colon tissue samples were homogenized with a Dounce homogenizer in a cold buffer containing 250 mM sucrose, 3 mM EDTA and 1 mM DTT at pH 7.4 supplemented with a cocktail of protease inhibitors (Sigma-Aldrich, St. Louis, MO, USA). Once samples appeared to be homogeneously disrupted, homogenates were centrifuged at 1000× *g* for 10 min at 40 °C. The supernatant containing the cytosolic fraction was transferred into a separate 1.5 mL Eppendorf tube and kept on ice, while centrifugation was repeated a second time to wash nuclei pellets. Then, pellets were resuspended in RIPA buffer supplemented with a cocktail of protease and phosphatase inhibitors (Sigma-Aldrich, St. Louis, MO, USA) and sonicated (10 sec.) to ensure proper homogenization. The protein concentration of nuclei and cytosol fractions was determined as described above for total homogenates. Western blotting was used to determine the protein expression of transcription factors (AP-1, ATF6, IRE1, NF-κB, NRF2, PERK, p-PERK and p-SAPK) in nuclear samples, whereas IκB and SAPK/JNK protein expression was assessed in cytosol samples. 

### 2.8. Glutathione Peroxidase (GPx) Activity

Liver and colon tissue samples were homogenized in PBS buffer, and supernatant proteins were obtained after centrifugation at 10,000× *g* for 5 min at 4 °C to determine glutathione peroxidase activity by Elisa kits (ABCAM, Cambridge, UK). 

### 2.9. Free Fatty Acid Analysis

Free fatty acid determination was performed in liver tissue. For liver sample preparation, 0.1 g of tissue was homogenized in 1 mL PBS-EDTA 4 mM, and lipids were extracted overnight at 4 °C. After evaporation of the lower phase, lipids were resuspended in 600 μL of H_2_O. Samples were then subjected to transesterification and injected into a gas chromatograph (Agilent 7890 GC, Mississauga, ON, Canada) using a 90 m × 0.32 mm WCOT-fused silica capillary column VF-23ms coated with 0.25 μm film thickness (Agilent, Mississauga, ON, Canada), as previously described [33]. 

### 2.10. Metagenomic DNA Extraction

Metagenomic DNA was extracted from feces and cecal scrapings using the MP FastDNA SPIN kit and FastPrep-24 instrument (MP Biomedicals, Santa Ana, CA, USA), as previously described [34]. Briefly, samples were subjected to two rounds of mechanical lysis at 6 m/s for 40 s, with cooling on ice for at least 5 min between lysis rounds, followed by the standard manufacturer’s protocol. Metagenomic DNA was eluted in H_2_O and the DNA concentration in each sample was measured using a Qubit fluorimeter (Thermo Fisher Scientific, Waltham, MA, USA) as per the manufacturer’s protocol. Samples were normalized to 5 ng/µL prior to library construction.

### 2.11. 16S rRNA V6 Library Construction for Ion Torrent Sequencing and Analysis

V6-16S rRNA amplicon libraries were constructed and sequenced as previously described, with samples randomly distributed between sequencing libraries and selected samples sequenced multiple times to control for potential batch effects [34]. Briefly, the V6 hypervariable region of the extracted metagenomic DNA was amplified in the PCR reaction and each sample was pooled at equal masses. The amplicon pool was purified, size-selected and sequenced on an S5 Ion Torrent using 540 chips according to the manufacturer’s instructions. Raw sequencing reads were demultiplexed using cutadapt discarding reads without both universal primers, reads with ambiguous bases and reads <100 in length after primer removal [35]. These demultiplexed/filtered reads have been deposited to NCBI SRA under project accession PRJNA1016344. Amplicon sequence variants (ASVs) were identified using the dada2 workflow [36] and subsequent analysis was performed using phyloseq [37]. Briefly, reads were quality-filtered to remove those with expected errors >1 and the error profile was determined using the recommended settings for Ion Torrent datasets. Reads were denoised and chimera filtered in dada2 with sample pooling. Taxonomy was assigned using the RDP Naive Bayesian Classifier algorithm using the default parameters against the Silva 132 database. The resulting ASVs were inserted into the Silva 132 backbone tree using SEPP with the default settings to calculate phylogenetic distances [38]. ASV functional potentials were determined using PICRUSt2 [39].

### 2.12. Short Chain Fatty Acid Analysis

Initial (week 0) and final (week 11) stools from mice in each group were analyzed for total SCFA content (acetic, propionic, butyric, isobutyric, 2-methyl butyric, isovaleric, 2-methyl valeric, caproic and isocaproic acid). For each time point, 2–3 mouse stool pellets were precisely weighed, and 50% aqueous acetonitrile (10 μL per μg) was added. The samples were homogenized using FastPrep-5G and subsequently centrifuged at 16,000× *g* at 10 °C for 10 min and the supernatant was removed. The levels of SCFAs were quantified using a derivatization method, as published in a previous study [40]. Briefly, the 40 μL supernatant was reacted with 20 μL of 200 mM 3-nitrophenylhydrazine (3NPH) and 20 μL of 120 mM of N-(3-dimethylaminopropyl)-N′-ethylcarbodiimide hydrochloride-6% pyridine solution at 40 °C for 30 min. The samples were diluted with 10% aqueous acetonitrile and mixed with ^13^C_6_-3NPH-labeled SCFAs as internal standards. The mixtures were analyzed on an Orbitrap Exploris 480 mass spectrometer (Thermo Fisher Scientific, Waltham, MA, USA) in PRM mode equipped with a tip column (75 μm inner diameter ×15 cm) packed with reverse phase beads (3 μm/120 Å ReproSil-Pur C18 resin, Dr. Maisch HPLC GmbH, Ammerbuch, Germany). The concentrations of SCFAs were calculated based on the standard calibration curve using internal standards. Samples with values below the quantitative limit of the assay were discarded from further analysis and SCFAs with >90% of the samples below the detection limit were also removed (2-methyl valeric, caproic and isocaproic acids).

### 2.13. General Statistical Analysis

All values were expressed as the mean ± SEM. Data were either analyzed by two-way ANOVA when temporal evolution was examined or by one-way ANOVA followed by Tukey’s multiple comparisons test. All analyses were performed using PRISM 7.0 (GraphPad Software, San Diego, CA, USA). Differences were considered significant at *p* ≤ 0.05. A frequency distribution test was performed on adipocytes cell area, and a one-way analysis of variance (ANOVA) followed by a Tukey’s multiple comparisons test was used to compare adipocyte area among groups. 

### 2.14. Microbiome Statistical Analysis

For the 16S amplicon data analysis, contaminant ASVs were first identified and removed with decontam using the frequency method to control for the sequencing batch [41]. ASVs annotated as eukaryota, chloroplast or mitochondria were also removed as likely off-target amplicons. ASVs were subsequently filtered to only keep those with ≥2 counts in at least 5% of the final sample dataset, and samples with <39,000 reads after filtering were removed from further analysis. Where required, a pseudocount was added to allow for plotting log10-transformed relative abundances. Technical replicates were merged at equal ratios and samples were rarefied to 39,000 reads where appropriate. Differences in alpha diversity were assessed with the Chao1 (richness) and Shannon (evenness) indices using Mann–Whitney or Kruskal–Wallis tests as appropriate. Sample beta diversity was examined using the weighted Unifrac distance and significant clustering was assessed using PERMANOVA via the adonis2 function in vegan [42] (Supplementary Data, Table S3). Differentially abundant ASVs and functional potential between Bipro and GMP mice at the end of the experiment were determined by analyzing microbiota communities in the cecal samples and using the final collected stools using both meteagenomeSeq and DESeq2 [43,44]. ASVs/functions were considered differentially abundant if detected by both algorithms with a fold change ≥1.5 and an adjusted *p* value ≤ 0.05 (Supplementary Data, Table S4). MetagenomeSeq’s fitTimeSeries function was used to identify ASVs/functions with different abundance patterns over time between the Bipro and GMP mice. Multiple comparisons were controlled using the Benjamini and Hochberg approach and the criterion for statistical significance was set at *p* < 0.05 with a differential abundance interval length > 1 week (Supplementary Data, Table S5). 

## 3. Results

### 3.1. GMP Supplementation Prevents Excessive Weight Gain and Adipocyte Hypertrophy

**Figure 1:**
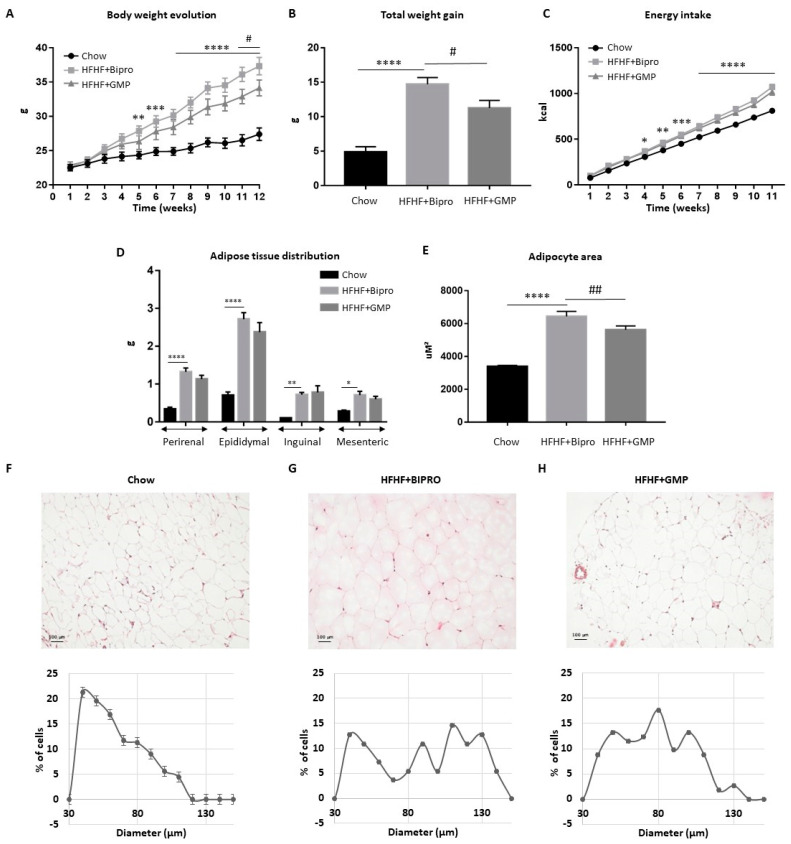
Glycomacropeptide supplementation attenuated high-fat, high-fructose diet-induced obesity in mice without impacting food intake. Mice were fed either a standard chow or a high-fat, high-fructose diet (HFHF) for 12 weeks. Chow-fed animals were administered daily a water vehicle while HFHF diet-fed animals received oral doses of Bipro (HFHF + Bipro) or glycomacropeptide (HFHF + GMP) (200 mg/kg). (**A**) Body weight evolution. (**B**) Total weight gain. (**C**) Energy intake. (**D**) Fat pad distribution (perirenal, epididymal inguinal and mesenteric fat pads) (*n* = 8/group). At the end of the 12-week experiment, mesenteric adipose tissues were fixed, paraffin-embedded and stained with HPS. Representative images of (**F**) chow-fed mice, (**G**) HFHF + Bipro-fed mice and (**H**) HFHF + GMP-fed mice adipocytes were taken (magnification × 200, scale bar = 100 μm). Adipocyte size distribution (%) was then calculated for (**F**) chow, (**G**) HFHF + Bipro and (**H**) HFHF + GMP groups as well as (**E**) adipocyte area (μM^2^) (*n* = 4/group). Data are expressed as the mean ± SEM. Versus chow: * *p* < 0.05, ** *p* < 0.01, *** *p* < 0.001, **** *p* < 0.0001; versus HFHF + Bipro: ## *p* < 0.01, # *p* < 0.05.

To determine the effect of GMP on diet-induced obesity, mice were fed with either a chow or HFHF diet for 12 weeks. Of note, the administration of Bipro or GMP did not result in any adverse event during the 12-week period. As shown in Figure 1A, administration of the HFHF diet from week 4 to week 12 resulted in a significant increase in body weight compared to the chow group. Although total energy intake was similar between the HFHF + Bipro and HFHF + GMP groups (Figure 1C), GMP supplementation resulted in lower body weight increment and total body weight gain (Figure 1A,B). The difference in body weights between the HFHF + Bipro and HFHF + GMP groups did not appear to derive from differences in perirenal, epididymal, inguinal and mesenteric adipose tissue (Figure 1D). Given the importance of mesenteric adipose tissue in metabolic health, we compared histopathological HPS staining in mesenteric tissues of chow, HFHF + Bipro and HFHF + GMP animals (Figure 1E–H). The calculation of adipocyte cell diameter distribution revealed marked differences among groups. Chow-fed mice displayed the highest proportion (≈22%) of small adipocytes (≈40 μm), with a total absence of adipocytes larger than 120 μm in comparison to HFHF diet-fed mice (Figure 1F). In contrast, HFHF diet-fed mice displayed a low percentage (≈13%) of small adipocytes and a higher proportion (≈14%) of adipocytes ranging from 120 μm to 150 μm (Figure 1G). Interestingly, GMP supplementation led to an adipocyte distribution between chow and HFHF (Figure 1H), suggesting an ameliorated adipose tissue profile. Accordingly, mean adipocyte cell area significantly differed between chow, HFHF + Bipro and HFHF + GMP animals (3385 μm^2^ ± 74.34, 6438 μm^2^ ± 305.5 and 5627 μm^2^ ± 227.2, respectively) (Figure 1E).

### 3.2. GMP Supplementation Alleviates Systemic Insulin Resistance and Inflammation

**Figure 2:**
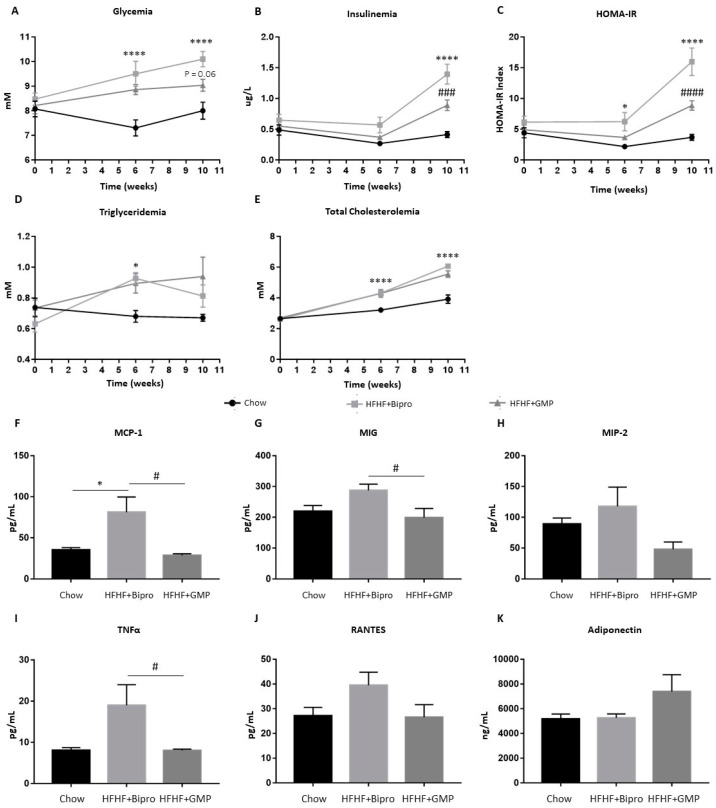
Glycomacropeptide administration prevented systemic insulin resistance and inflammation in high-fat, high-fructose diet-fed mice. At weeks 0, 6 and 10, blood samples were obtained after a 12 h overnight fast to assess insulin resistance and dyslipidemia development in mice (*n* = 8/group). (**A**) Glycemia, (**B**) insulinemia and (**C**) homeostatic model assessment of insulin resistance (HOMA-IR), (**D**) triglyceridemia and (**E**) total cholesterolemia. At the end of the 12-week experiment, plasma was collected to characterize mouse inflammatory profiles (*n* = 8/group). The following cytokines were found to be modulated by GMP treatment: (**F**) MCP-1, (**G**) MIG, (**H**) MIP-2, (**I**) TNF-α and (**J**) RANTES. (**K**) Adiponectin concentration was also determined as described in the Materials and Methods section. Data are expressed as the mean ± SEM. Versus chow: * *p* < 0.05, **** *p* < 0.0001; versus HFHF + Bipro: # *p* < 0.05, ### *p* < 0.001, #### *p* < 0.0001. MCP-1: monocyte chemoattractant protein; MIG: monokine induced by gamma interferon; MIP-2: macrophage inflammatory protein; TNF-α: tumour necrosis factor alpha; RANTES: regulated on activation, normal T cell expressed and secreted.

As insulin insensitivity is a major feature of MetS and usually results in elevated insulin production to lower circulating glucose concentrations, we examined glycemia and insulinemia in all three groups of fasting animals. High levels of glucose and insulin were observed in response to HFHF throughout the experiment. However, GMP administration reduced the magnitude of this increase. A maximal beneficial action was observed at week 10 (Figure 2A,B). The calculation of the HOMA-IR index confirmed an improvement of insulin sensitivity by GMP, with the utmost impact at week 10 (Figure 2C). As noted in Figure 2D,E, mice on the HFHF diet developed hypertriglyceridemia and hypercholesterolemia compared to mice on the chow diet, with no significant changes upon treatment with GMP. 

As low-grade inflammation is intrinsically linked to MetS development, we analyzed the concentrations of various cytokines, chemokines and inflammation-related proteins in the plasma of mice sacrificed at week 12. As expected, the Bio-Plex technology revealed that HFHF + Bipro mice, compared to chow-fed mice, displayed higher levels of pro-inflammatory cytokines and chemokines and a complex of network molecules that regulate cell proliferation, differentiation and survival (Supplementary Data, Figure S2). These results reflect a higher state of inflammation in response to HFHF feeding. Strikingly, GMP supplementation lowered various pro-inflammatory cytokines (i.e., MCP-1, MIG and TNFα) known to be implicated in MetS development to a level comparable to or even lower than that of chow-fed mice (Figure 2F–J). These results suggest that HFHF diet-induced systemic inflammation could be harnessed by GMP administration. We next determined the plasma concentration of adiponectin, a protein secreted by adipose tissue, endowed with anti-inflammatory properties (Figure 2K). Although adiponectin levels were not statistically different between groups, the observed mean in HFHF + GMP was 40% higher than in HFHF + Bipro mice. 

### 3.3. GMP Impact on Gut Morphology and Permeability

**Figure 3:**
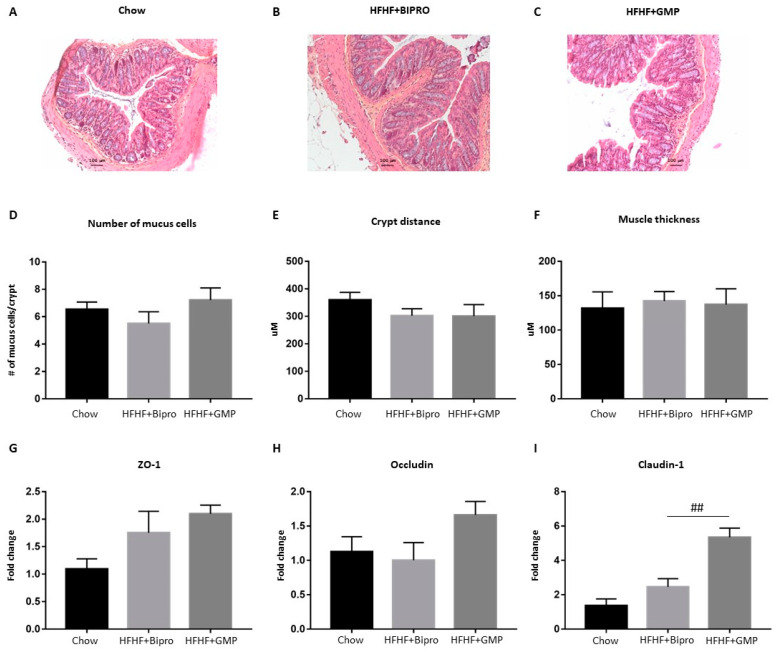
Impact of glycomacropeptide treatment on intestinal morphology and permeability. Cross-sections of distal colon tissue were stained with hematoxylin and eosin (H&E) to evaluate GMP’s impact on intestinal morphology. Representative images of colon sections of (**A**) chow, (**B**) HFHF + Bipro and (**C**) HFHF + GMP mouse groups are presented (magnification  ×  200, scale bars = 100 μm). Histological parameters including (**D**) number of mucus cells per crypt, (**E**) crypt distance and (**F**) muscle thickness. Data are shown as the mean ± SEM. Gene expression of tight junction proteins (**G**) ZO-1, (**H**) occludin and (**I**) claudin-1 was determined by RT-qPCR, as described in the Materials and Methods section. Data are expressed as the mean ± SEM (*n* = 4–8/group). Versus HFHF + Bipro: ## *p* < 0.01. ZO-1: zonula occludens-1.

Colon sections were stained with HES for histologic assessment (Figure 3A–C). Apart from a slight trend toward a decrease in the number of mucus cells in response to the HFHF diet, there was little change in the distance between colonic crypts or muscle thickness compared to chow-fed animals. (Figure 3D–F). Likewise, colonic tissues from GMP-treated animals exhibited a subtle but nonsignificant increase in mucus cells, which may indicate partial tissue recovery (Figure 3D). 

We then turned to the analysis of zonula occludens (ZO)-1, occludin and claudin-1, which play a crucial role in maintaining the integrity and function of the intestinal barrier (Figure 3G–I). GMP administration resulted in a slight tendency to increase ZO-1 and occludin, but significantly upregulated claudin-1 gene expression (Figure 3H,I). 

### 3.4. GMP Fights Gut Inflammation, Abnormal Redox State and ER Stress

**Figure 4:**
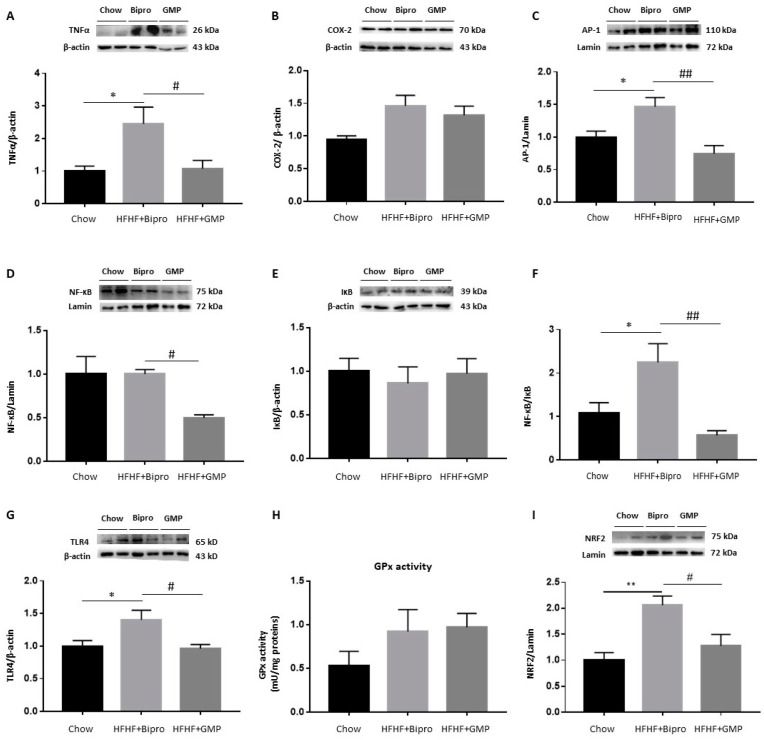
Effects of glycomacropeptide on intestinal inflammation and oxidative stress. Protein levels of key inflammation and antioxidant defence biomarkers were evaluated in the distal colon of mice by Western blot: (**A**) TNF-α, (**B**) COX-2, (**C**) AP-1, (**D**) NF-κB, (**E**) IκB, (**G**) TLR4 and (**I**) NRF2 as described in the Materials and Methods section, and the NF-κB/IκB ratio was calculated (**F**). AP-1 and NF-κB proteins were assayed on the same membrane using their specific antibodies and shared the same reference protein lamin. (**H**) GPx activity was determined by enzymatic kit as described in the Materials and Methods section. Data are expressed as the mean ± SEM (*n* = 4/group). Versus chow: * *p* < 0.05, ** *p* < 0.01; versus HFHF + Bipro: # *p* < 0.05, ## *p* < 0.01. TNF-α: tumor necrosis factor alpha; COX: cyclooxygenase; AP: activator protein; NF- κB: nuclear factor-kappa B; IκB: inhibitor of kappa B; TLR4: toll-like receptor 4; NRF2: nuclear factor erythroid 2-related factor 2; GPx: glutathione peroxidase.

Since intestinal inflammation, OxS and ER stress are linked to MetS development, it was necessary to define their status and examine the effect of GMP. Inflammation in the distal colon was appraised by evaluating the protein levels of TNF-α, COX-2, AP-1, NF-κB and IκB by Western blotting (Figure 4A–E). HFHF + Bipro-fed mice displayed raised protein levels of TNF-α and COX-2, two powerful inflammatory mediators in the distal colon, compared to chow-fed animals (Figure 4A,B). Levels of the potent transcription factor AP-1 and the NF-κB/IκB ratio were also found to be elevated in HFHF + Bipro colon tissue (Figure 4C,F). However, GMP supplementation resulted in decreased TNF-α protein expression while lowering AP-1 and the NF-κB/IκB ratio to chow-fed mice levels. We also analyzed the protein expression of TLR4 as it is the receptor that specifically recognizes lipopolysaccharide (LPS) from the outer membrane of Gram-negative bacteria. As noted in Figure 4G, there was a significant increase in TLR4 protein expression in the colon of HFHF + Bipro mice, but GMP brought it back to chow-fed mice levels. When we assessed the protein expression of NRF2, the transcription factor that functions as a regulatory agent for the genes involved in the antioxidant defence, we found higher levels in HFHF + Bipro colon tissue compared to chow-fed animals and a normalization of the transcription factor protein expression by GMP (Figure 4I). No significant changes were observed in GPx activity (Figure 4H).

**Figure 5:**
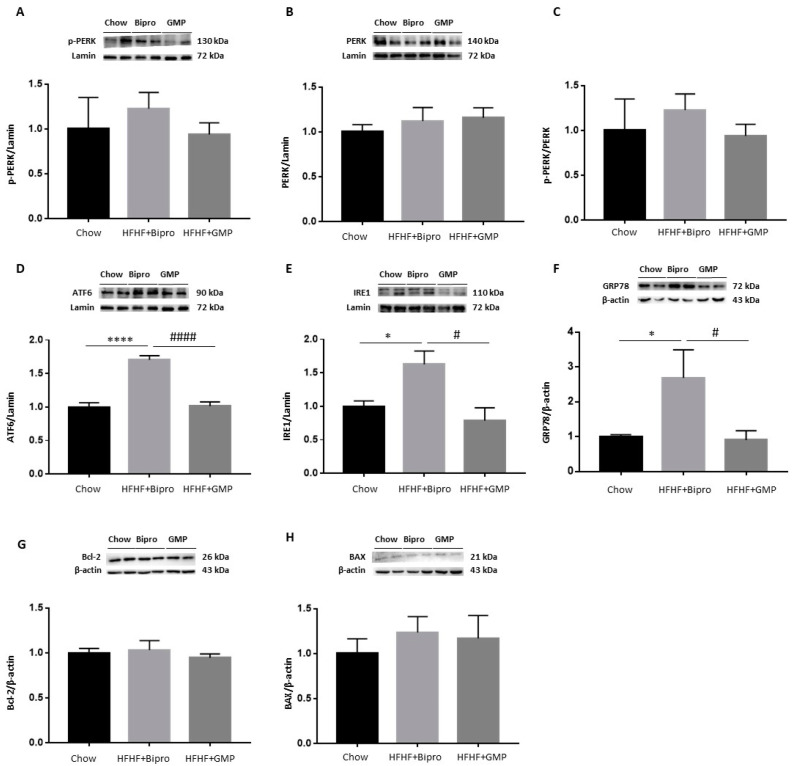
Glycomacropeptide alleviated intestinal endoplasmic reticulum stress in high-fat, high-fructose diet-treated animals. Protein expression of endoplasmic reticulum (ER) stress markers (**A**) p-PERK, (**B**) PERK, (**D**) ATF6, (**E**) IRE1, (**F**) GRP78, (**G**) Bcl-2 and (**H**) BAX was assessed by Western blot in the distal colon of mice and the (**C**) p-PERK/PERK ratio was calculated. p-PERK and ATF6 proteins were assayed on the same membrane using their specific antibodies and shared the same reference protein lamin. Data are expressed as the mean ± SEM (*n* = 4/group). Versus chow: * *p* < 0.05, **** *p* < 0.0001; versus HFHF + Bipro: # *p* < 0.05, #### *p* < 0.0001. PERK: phospho protein kinase RNA-like ER kinase; ATF6: activating transcription factor 6; IRE1: inositol-requiring enzyme 1; GRP78: glucose-regulated protein 78; Bcl-2: B-cell lymphoma 2; BAX: Bcl2-associated X protein.

To examine ER stress, we first determined the protein expression of PERK, ATF6 and IRE1, each initiating a specific branch of the unfolded protein response (UPR). Western blot analysis revealed invariant results for PERK and *p*-PERK (Figure 5A,B), but a significant elevation of ATF6 and IRE1 in HFHF + Bipro distal colon tissue (Figure 5D,E). Although the calculated p-PERK/PERK ratio followed the same trend, it was not significant (Figure 5C). Similarly, raised protein expression of GRP78 was observed in the distal colon in response to HFHF + Bipro feeding (Figure 5F). In turn, GMP exhibited a great ability to normalize these ER parameters to the level of chow-fed mice, thereby reducing the heavy load on the ER. Because ER stress can also activate apoptotic pathways, we focused on the status of the pro-apoptotic BAX and the anti-apoptotic Bcl-2 proteins, which are key regulators of the intrinsic apoptotic pathway. No marked variation was observed in the expression of BAX or Bcl-2 following HFHF feeding or GMP treatment (Figure 5G,H). 

### 3.5. Attenuation of Intestinal Insulin Resistance by GMP

**Figure 6:**
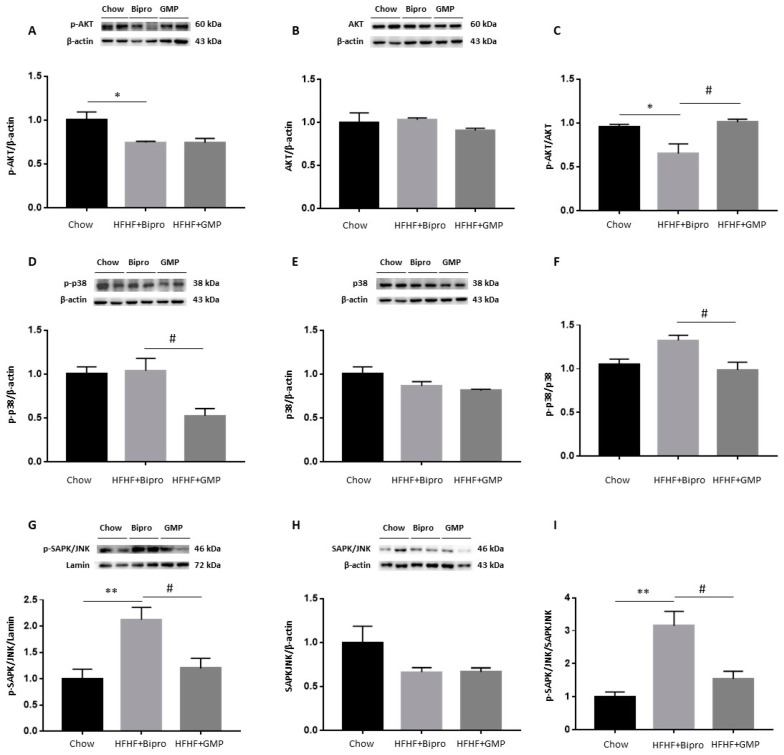
Glycomacropeptide increases gut insulin sensitivity, modulates insulin signalling and downregulates mitogen-activated protein kinases. Protein expression of important biomarkers involved in the insulin signalling cascade was determined by Western blot in mice colons: (**A**) phospho-AKT, (**B**) AKT, (**D**) phospho p38-MAPK, (**E**) p38-MAPK, (**G**) phospho SAPK//JNK and (**H**) SAPK/JNK. The ratios of (**C**) phospho AKT/AKT, (**F**) phospho p38-MAPK/p38-MAPK and (**I**) phospho SAPK-JNK/SAPK-JNK were then calculated. AKT and p38 proteins were assayed on the same membrane using their specific antibodies and shared the same reference protein β-actin. Results represent the mean ± SEM (*n* = 4 /group). Versus chow: * *p* < 0.05, ** *p* < 0.01; versus HFHF + Bipro: # *p* < 0.05. MAPK: mitogen-activated protein kinase; SAPK: stress-activated protein kinase; JNK: jun amino-terminal kinase.

Given the close association between inflammation, OxS, ER stress and IR, we determined the protein expression of AKT, a key protein involved in the insulin signalling pathway. While no change was detected in the expression of total AKT (Figure 6B), its phosphorylation was diminished by HFHF feeding (Figure 6A), which led to a reduced p-AKT/AKT ratio, reflecting a decline in insulin sensitivity in the distal colon of HFHF + Bipro-treated mice (Figure 6C). However, treatment with GMP led to an increase in the p-AKT/AKT ratio, indicating the restoration of insulin sensitivity (Figure 6A–C). We also determined the protein levels of p38 MAPK and SAPK/JNK, which are greatly implicated in IR development. Although no differences were detected in total SAPK/JNK and p38 MAPK total protein mass among the experimental groups (Figure 6E,H), the expression of their phosphorylated forms was significantly lessened by GMP (Figure 6D,G), leading to lower p-p38 MAPK/p38 MAPK and p-SAPK/SAPK ratios in the HFHF + GMP group (Figure 6F,I). Since p-p38 MAPK is known to phosphorylate ATF6 and lead to UPR in several pathophysiological models [45], the reduction in intestinal ER stress observed in our study may be partly related to the reduction in the phosphorylation of p38 MAPK by GMP.

### 3.6. Bile Acid Metabolism Shift in Gut–Liver Axis in Response to GMP Administration

**Figure 7:**
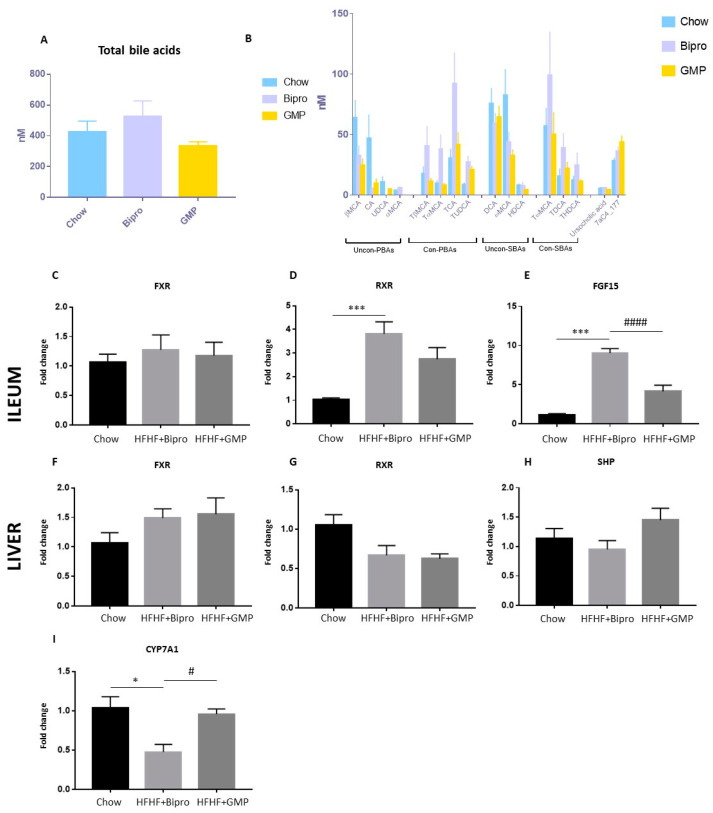
Impact of glycomacropeptide on bile acid metabolism in the gut–liver axis. (**A**) Bile acid pool size and (**B**) composition were assessed in mice plasma as described in the Materials and Methods section. Messenger RNA was extracted from the ileum and liver and gene expression of (**C**,**F**) FXR, (**D**,**G**) RXR, (**E**) FGF15, (**H**) SHP and (**I**) CYP7A1 was determined by RT-qPCR, as described in the Materials and Methods section. Results represent the mean ± SEM (*n* = 4–8/group). Versus chow: * *p* < 0.05, *** *p* < 0.001; versus HFHF + Bipro: # *p* < 0.05, #### *p* < 0.0001. FXR: farnesoid X receptor; RXR: retinoid X receptor; FGF15: fibroblast growth factor 15; SHP: small heterodimer partner; CYP7A1: cholesterol 7α-hydroxylase.

As bile acids (BAs) serve as critical signalling molecules connecting distant tissues via blood circulation, particular attention has been devoted to the gut–liver axis and intrinsic transcription factors that do not only regulate bile acid transport metabolism but also glucose and lipid metabolism. A trend toward higher BA production was observed in the HFHF diet-fed group compared to the chow-fed group, and a trend toward the restoration of BA levels was observed with GMP supplementation (Figure 7A). Accordingly, we observed some changes in specific categories of BA, but no significant differences between groups (Figure 7B). We therefore examined the gene expression of farnesoid X receptor (FXR), retinoid X receptor (RXR) and fibroblast growth factor 15 (FGF15) in the ileum, which represents the major site of BA recycling. No significant changes were noted in FXR, whereas a marked upregulation was observed in RXR and FGF15 mRNA expression in response to HFHF + Bipro treatment (Figure 7C–E). Interestingly, GMP reversed the trend by preventing the HFHF diet-induced increase in RXR and FGF15. A completely different profile of these key factors characterized the liver: a trend in the increase of FXR gene expression and a decrease of RXR was observed whether in the presence of HFHF + Bipro or HFHF + GMP (Figure 7F,G). Accordingly, an increasing trend was also apparent in the gene expression of the FXR small heterodimer partner (SHP) in response to GMP (Figure 7H). HFHF + Bipro mice showed lower levels of cholesterol 7α-hydroxylase (CYP7A1), an enzyme that catalyzes the conversion of cholesterol into primary BA, while HFHF + GMP mice displayed gene expression levels comparable to those of chow-fed mice (Figure 7I). 

### 3.7. Impact of GMP on NAFLD

**Figure 8:**
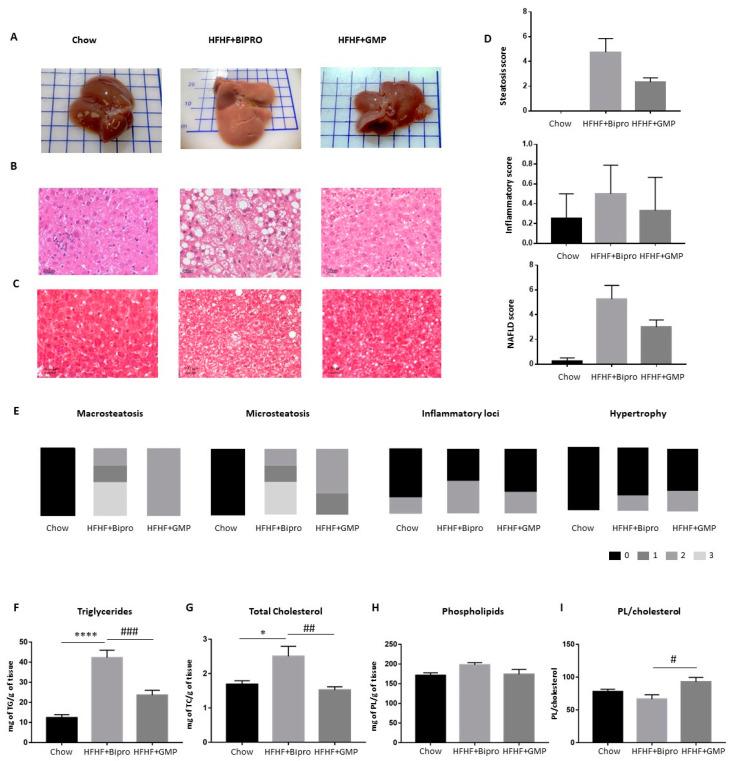
Supplementation with glycomacropeptide reduced the severity of nonalcoholic fatty liver disease in high-fat, high-fructose diet-fed mice. At the end of 12 weeks, (**A**) liver pictures of chow-fed mice, HFHF diet-fed mice supplemented with Bipro and HFHF diet-fed mice supplemented with GMP were taken. After fixation and embedding (parafilm), the hepatic tissues were either stained with (**B**) HPS or (**C**) Masson’s trichrome and representative histological images were taken (HPS: magnification × 630, scale bar = 100 μm; trichrome: magnification × 200, scale bar= 100 μm). Based on these images, (**D**) steatosis, inflammatory and NAFLD scores were calculated. (**E**) The graphs summarize the severity of macrosteatosis, microsteatosis, inflammatory loci and hypertrophy in each group based on Liang’s scoring system. (**F**) Triglycerides, (**G**) total cholesterol and (**H**) phospholipid content in the liver were quantified as described in the Materials and Methods section. (**I**) Then, the phospholipid/cholesterol ratio was calculated. Results represent the mean ± SEM (*n* = 4/group). Versus chow: * *p* < 0.05, **** *p* < 0.0001; versus HFHF + Bipro: # *p* < 0.05, ## *p* < 0.01, ### *p* < 0.001. HPS: hematoxylin-phloxin saffron; NAFLD: nonalcoholic fatty liver disease; PL: phospholipid.

Since HFHF feeding promotes hepatic fat deposition and the development of NAFLD, histopathology based on HPS and Masson’s trichrome staining as well as biochemical analyses were performed to assess liver status. Liver pictures, histopathological HPS and Masson’s trichrome staining demonstrated clear differences in lipid droplet size and number between chow, HFHF + Bipro and HFHF + GMP animals (Figure 8 A–C). As a matter of fact, the mean steatosis score was 4.75 for HFHF + Bipro compared to 2.33 for the HFHF + GMP group (Figure 8D). Compared to chow-fed animals, HFHF diet-fed mice displayed a higher inflammatory score that was substantially restrained by GMP treatment. A combination of both indices to calculate the NAFLD index revealed a marked attenuation of the score (5.25 versus 3) following treatment with GMP. Figure 8E details histological parameters used in NAFLD scoring, including macrosteatosis, microsteatosis, inflammatory loci and hepatocyte hypertrophy. It is noteworthy that a greater proportion of HFHF + Bipro mice displayed scores of 2 and 3 when evaluated for micro and macrosteatosis, whereas a majority of HFHF + GMP mice presented with scores of 1 or 2, suggesting less severe fat deposition. 

Biochemical measurements of liver TG and TC concentrations revealed a significant effect of GMP on hepatic TG and TC content (Figure 8F,G). Given the importance of phospholipid content to maintain cell membrane fluidity, we assessed its levels in the liver. Total phospholipid concentrations did not significantly differ between groups (Figure 8H), but the phospholipid/cholesterol ratio was significantly higher in the HFHF + GMP group compared to the HFHF + Bipro group (Figure 8I), suggesting a beneficial effect of GMP treatment on hepatic membrane fluidity. In line with the alleviation of hepatic steatosis, GMP animals exhibited lower concentrations of total, saturated, mono-unsaturated, *n*-7 and *n*-9 fatty acids compared to Bipro animals. Accordingly, we estimated the activity of SCD1-Δ9, as well as the lipogenesis index, respectively, by comparing the ratios of 18:1(*n*-9)/18:0 and C16:0/C18:2 (*n*-6), and found that both were reduced in the GMP group (Supplementary Data, Table S6).

### 3.8. GMP Attenuates Hepatic Inflammation and OxS

**Figure 9:**
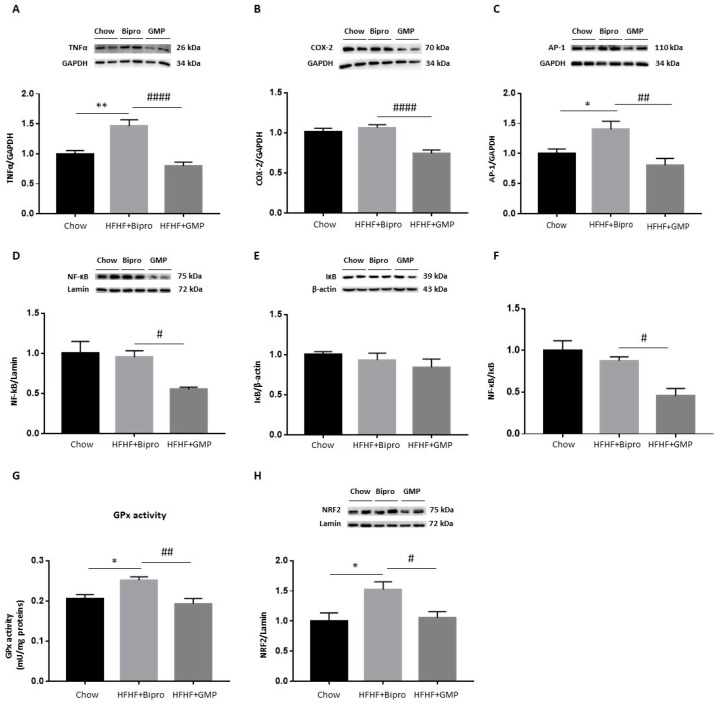
Glycomacropeptide reduced hepatic inflammation and oxidative stress. Protein expression of key inflammation and antioxidant defence biomarkers were evaluated in mouse livers by Western blot: (**A**) TNF-α, (**B**) COX- 2, (**C**) AP-1, (**D**) NF-κB, (**E**) IκB and (**H**) NRF2, as described in the Materials and Methods section. The NF-κB/IκB ratio was then calculated (**F**). COX-2 (Figure 9B) and SAPK/JNK (Figure 11E) proteins were assayed on the same membrane using their specific antibodies and shared the same reference protein GAPDH. (**G**) GPx activity was determined by enzymatic kit. Data are expressed as the mean ± SEM (*n* = 4–8/group). Versus chow: * *p* < 0.05, ** *p* < 0.01; versus HFHF + Bipro: # *p* < 0.05, ## *p* < 0.01, #### *p* < 0.0001. TNF-α: tumour necrosis factor alpha; COX: cyclooxygenase; AP: activator protein; NF-κB: nuclear factor-kappa B; IκB: inhibitor of kappa B; NRF2: nuclear factor erythroid 2-related factor 2; GPx: glutathione peroxidase.

Similar to what was observed in the distal colon, GMP strongly attenuated inflammation in the liver, as evidenced by reduced protein expression levels of TNF-α, COX-2, AP-1 and NF-κB (Figure 9A–D). The calculation of the NF-κB/IκB ratio using NF-κB and IκB protein expression results confirmed this trend (Figure 9D–F). As for OxS status in the liver, HFHF + Bipro feeding increased GPx activity, while GMP supplementation restored it to chow-fed mice levels (Figure 9G). Following the same trend as GPx activity, Western blotting revealed a strong activation of NRF2 by HFHF + Bipro feeding, which was neutralized by GMP supplementation (Figure 9H). 

### 3.9. Regulation of ER Stress and Apoptosis in the Liver by GMP

**Figure 10:**
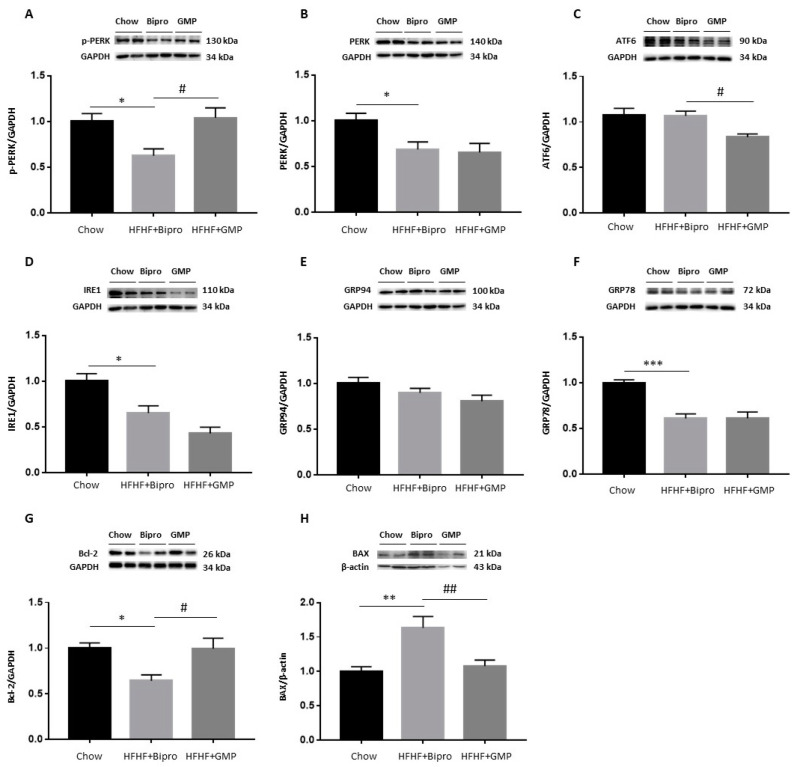
Glycomacropeptide did not prevent endoplasmic reticulum stress in the liver but averted its transition into apoptosis. Protein expression of endoplasmic reticulum (ER) stress markers (**A**) p-PERK, (**B**) PERK, (**C**) ATF6, (**D**) IRE1, (**E**) GRP94, (**F**) GRP78, (**G**) Bcl-2 and (**H**) BAX was assessed by Western blot in hepatic tissues. p-PERK and IRE1; PERK and GRP94; ATF6, GRP78 and p38 (Figure 11B), respectively, were assayed on the same membrane using their specific antibodies and shared the same reference protein GAPDH. Data are expressed as the mean ± SEM (*n* = 4–8/group). Versus chow: * *p* < 0.05, ** *p* < 0.01, *** *p* < 0.001; versus HFHF + Bipro: # *p* < 0.05, ## *p* < 0.01. PERK: phospho protein kinase RNA-like ER kinase; ATF6: activating transcription factor 6; IRE1: inositol-requiring enzyme 1; GRP: glucose-regulated protein; Bcl-2: B-cell lymphoma 2; BAX: Bcl2-associated X protein.

As ER stress plays a key role in the pathogenesis of NAFLD, liver protein expression of PERK, ATF6 and IRE1 was evaluated by Western blot (Figure 10B–D). The protein expression of GRP78 and GRP94, two major chaperones involved in the initiation of UPR in the liver, was also assessed (Figure 10E,F). HFHF + Bipro feeding resulted in a reduction of PERK and IRE1, as well as their main chaperone GRP78 (Figure 10B,D,F), while no change was detected in ATF6 or GRP94 protein expression (Figure 10C,E). Compared to HFHF + Bipro-fed mice, GMP-supplemented mice displayed increased levels of p-PERK (Figure 10A) and reduced levels of ATF6 (Figure 10C). 

Thereafter, we measured Bcl-2 and BAX protein expression. Bcl-2 was reduced while BAX expression was increased in HFHF livers, pointing toward the presence of apoptosis. But, more importantly, Bcl-2 and BAX protein concentrations were normalized by GMP supplementation.

### 3.10. Modulation of Insulin Signalling Cascade by GMP in the Liver

**Figure 11:**
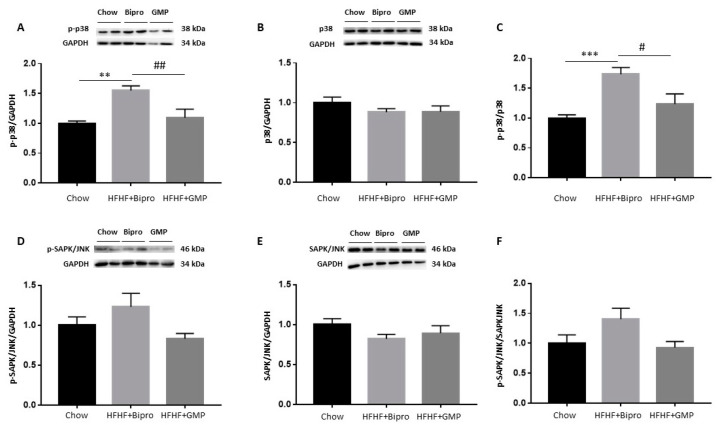
Modulation of insulin signalling in high-fat, high-fructose diet-fed mice livers by glycomacropeptide. Protein expression of major biomarkers involved in insulin signalling cascade was determined by Western blot in mice livers: (**A**) phospho p38-MAPK, (**B**) p38-MAPK, (**D**) phospho SAPK/JNK and (**E**) SAPK/JNK. The ratios of (**C**) phospho p38-MAPK/p38-MAPK and (**F**) phospho SAPK-JNK/SAPK-JNK were then calculated. Results represent the mean ± SEM (*n* = 4–8/group). Versus chow: ** *p* < 0.01, *** *p* < 0.001; versus HFHF + Bipro: # *p* < 0.05, ## *p* < 0.01. MAPK: mitogen-activated protein kinase; SAPK: stress-activated protein kinase; JNK: jun amino-terminal kinase.

We next investigated the modulation of several actors known to influence the insulin signalling cascade. As MAPK activity strongly affects insulin sensitivity, especially p38 MAPK and SAPK/JNK, we investigated their protein expression. While no difference was detected in total SAPK/JNK or p38 MAPK protein expression between groups (Figure 11B,E), their phosphorylation status was lessened by GMP (Figure 11A,D), leading to lower p-p38 MAPK/p38 MAPK and p-SAPK/SAPK ratios (Figure 11C,F).

### 3.11. HFHF Diet Dramatically Impacts Microbiota Composition, While GMP Has Subtle Effects on Microbial Composition

**Figure 12:**
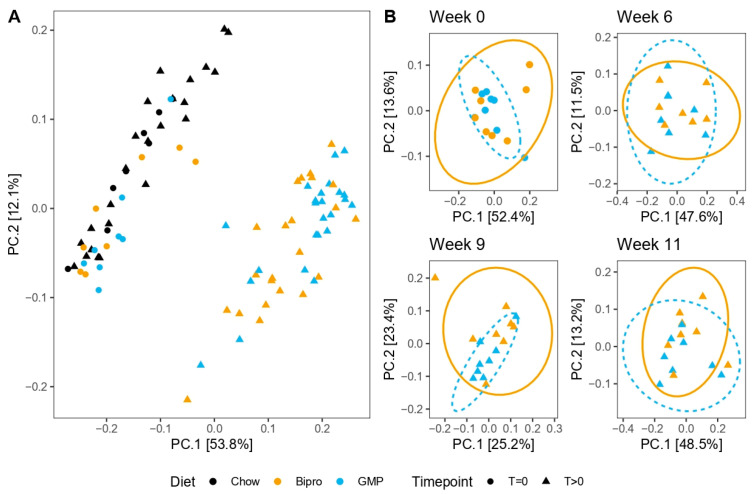
A high-fat, high-fructose diet dramatically shifted the microbiota composition over time regardless of glycomacropeptide supplementation. Principal coordinate analyses using the weighted Unifrac distance for all mouse stool samples (**A**) and the GMP/Bipro-fed mice at each sampling week (**B**). Ellipses represent the 95% confidence intervals for each group.

As the microbiota has been implicated in the deleterious impacts of MetS and is closely related to the various immunological processes that we observed to be impacted by HFHF feeding, we also characterized how it was impacted by GMP supplementation. The microbiotas from the chow, HFHF + Bipro and HFHF + GMP groups were indistinguishable at T0 when assessed using the weighted Unifrac distance (Supplementary Data, Table S3); however, the HFHF mice rapidly diverged from chow as the diet progressed (Figure 12A). Overall, the HFHF + Bipro and HFHF + GMP mice did not display overt differences in overall microbiota composition at any of the time points sampled (Figure 12B). 

**Figure 13:**
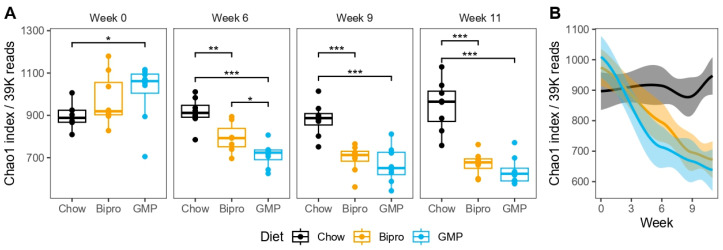
High-fat, high-fructose diet led to reduced species richness regardless of glycomacropeptide supplementation. Chao1 index between feeding groups at each sampling time point (**A**) and over time (**B**). Boxplots (**A**) show the interquartile range, with the median represented as a line. Changes over time (**B**) are represented as LOWESS fits to the data, with shaded regions denoting 95% confidence intervals. *p* < 0.5 *, 0.01 ** and 0.001 *** (note: only significant associations are plotted).

As expected, HFHF diet administration resulted in an overall reduction in species richness compared to the chow diet as species richness increased over time in the chow group (Figure 13). There was a trend for the HFHF + GMP mice to have lower richness compared to the HFHF + Bipro group, but this was only significant at week 6. 

In contrast, there were no differences in species evenness over time (Supplementary Data, Figure S3). Similar trends were observed in the cecal samples, with a clear separation between the chow and HFHF mice but not between the HFHF + Bipro and HFHF + GMP themselves, as well as an overall reduction in species richness (Supplementary Data, Figure S4). There was also a trend for lower species richness in the HFHF + GMP mice compared to both the chow and HFHF + Bipro mice, but this did not reach statistical significance.

**Figure 14:**
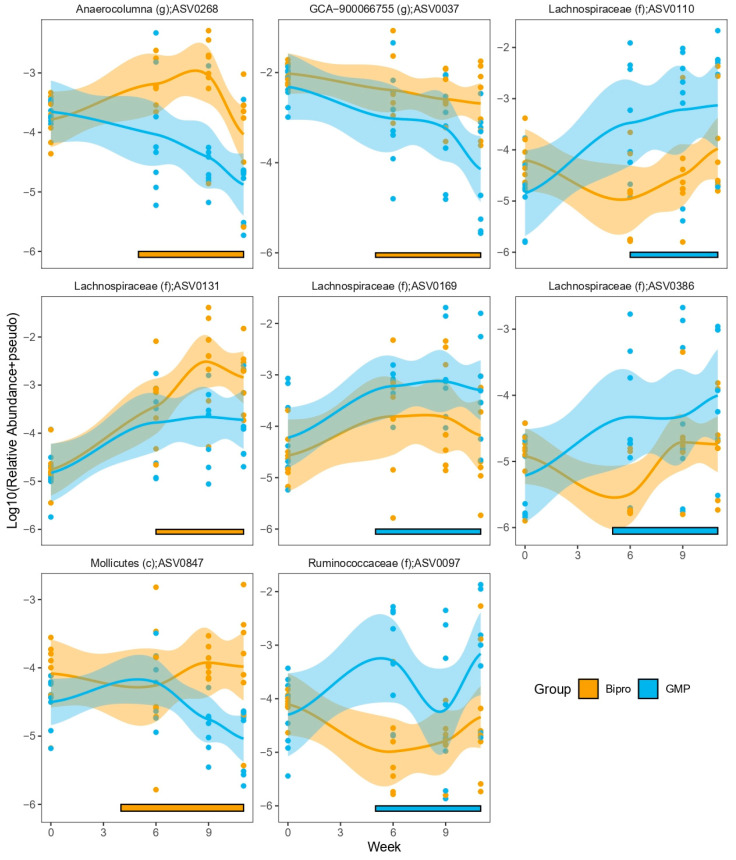
Glycomacropeptide had differing impacts on microbial abundance over time. Selected amplicon sequence variants (ASVs) identified as having differential abundance patterns over time. ASVs are shown with their lowest taxonomic annotation. ASV relative abundance was log10-transformed, with a pseudocount added as required. Points represent each sample, with lines representing LOWESS fits and shaded regions denoting 95% confidence intervals. Bars along the x-axis highlight the time spans identified as showing different abundance patterns, with the colour representing which group was enriched for each time period.

We next focused our analyses on identifying specific amplicon sequence variants (ASVs) that were differentially abundant between the HFHF + Bipro and HFHF + GMP mice to identify those that may be involved in modulating the differences seen between their host mice. Few ASVs were identified when simply assessing the final stool samples and cecal microbiotas (Supplementary Data, Table S4). However, we were able to identify several ASVs that showed differential abundance over time (Figure 14), with specific enrichment in either the HFHF + Bipro or HFHF + GMP groups (Supplementary Data, Table S5). In particular, there were numerous Lachnospiraceae family members that showed different tendencies to be enriched in either the HFHF + Bipro or HFHF + GMP groups. We also tested whether there were predicted functional differences between the groups either over time or at the end of the dietary intervention. However, we did not find any significant COG/KEGG/EC terms that appeared to differ in their predicted abundance based on 16S profiles.

As the microbiome can impact SCFA concentrations in the gut, we also assessed the levels of various SCFAs prior to and at the end of the intervention. There were no differences between the SCFAs in the mice groups prior to the dietary intervention (Supplementary Data, Figure S5). The chow mice showed minimal differences in their SCFAs over time, with only an increase in acetic acid noted at the end of the study. In contrast, both the HFHF + Bipro and HFHF + GMP mice showed increases in 2-methyl butyric and isovaleric acid, with the HFHF + GMP mice also showing a trend for increased isobutyric acid. There was a decrease in the levels of butyric acid between the chow and HFHF + Bipro mice, with no statistical difference between the chow and HFHF + GMP mice, although this could have been due to the increased variance seen in butyric acid levels in these animals (Supplementary Data, Figure S5). 

## 4. Discussion

Milk-derived peptides are gaining more and more popularity. The great interest of the scientific and medical community in these nutrients is explained by their potential to serve as natural alternatives to prevent and treat complex diseases. Bioactive milk proteins tend to have fewer adverse effects than pharmacological agents and are endowed with beneficial biological activities. This is precisely the case with GMP, considered to be one of the most promising bioactive milk molecules. Our recent study highlighted the protective effects of GMP in the context of Western diet consumption, including IR and hepatic metabolic disturbances in mice [13]. The aim of the present work was to further investigate the regulatory role and mechanisms of action of GMP in MetS. Collectively, our findings demonstrate the ability of GMP to combat obesity, counteract hyperglycemia and IR (by reducing OxS, inflammation and ER stress), alleviate liver steatosis (by downregulating TG and TC levels and modulating fatty acids profile), attenuate intestinal epithelial barrier dysfunction (by improving tight junction protein expression), and improve gut–liver axis homeostasis.

To increase the likelihood of drawing clear, reliable and generalizable conclusions from the present study, we strengthened the experimental conditions compared with the previous animal protocol [13]. The robust experimental design that was implemented included the addition of fructose to the drinking water to amplify the MetS, allowing the marked effect of GMP to be scrutinized. On the other hand, Bipro (an isocaloric control containing the same amino acids as GMP, but in a random order) was added to the HFHF group to better balance the caloric intake of GMP. The GMP dose of 200 mg/kg/day was selected based on previous metabolic studies of the effects of GMP in mice and rats [26,46,47,48].

Our first observation was that GMP played a critical role in controlling energy metabolism as it reduced weight gain by limiting adipocyte accretion during several weeks of hyperconsumption of a calorie-dense diet. The resulting reduction in weight gain underlines the potential of GMP to fight obesity, a major component of MetS [49,50]. This reduction in body weight gain is likely mediated by small reductions in perirenal, epididymal and mesenteric adipose tissue weights; however, a body composition analysis by dual X-ray absorptiometry scan would be required to draw conclusions. The higher proportion of small adipocytes and lower fraction of large adipocytes found in GMP-treated animals suggest restricted hypertrophy, which is in line with a recent study highlighting the inhibition of preadipocyte proliferation, differentiation and lipid accumulation by GMP [51]. Since obesity contributes to the onset of type 2 diabetes, NAFLD and atherosclerosis, reducing its extent with GMP may prove useful in slowing down these metabolic complications.

As expected, excess calories from refined carbohydrates and sugars caused elevated blood glucose and insulin levels, suggestive of IR in our animal model. The calculation of the HOMA-IR index confirmed this hypothesis. However, GMP supplementation significantly limited hyperglycemia and hyperinsulinemia, suggesting that these animals were better able to respond to insulin signals and more effectively control carbohydrate metabolism. With obesity, IR represents the most important trigger of MetS, thereby revealing the importance of GMP in improving tissue insulin sensitivity, decreasing adipogenesis and avoiding metabolic complications.

Given the ability of GMP to reduce IR, the next step was to investigate the underlying mechanisms of action. Inflammation represents one of the main predisposing factors for IR as low-grade inflammation interferes with insulin signalling through the release of inflammatory agents, including cytokines and adipokines by adipocytes and immune cells [52]. For example, TNF-α induces IR by triggering the NF-κB pathway [26,45,46] and activating JNK [47], impairing IRS-1 and Akt phosphorylation [53], which translates the insulin signal. Our hypothesis was confirmed as GMP was found to attenuate the elevation of cytokine concentrations (MCP-1 and TNF-α) associated with HFHF, in parallel with a decrease in the HOMA-IR index. Therefore, one of the mechanisms by which GMP may reduce IR may be by attenuating overall inflammation. Noteworthily, one of the pathways targeted by GMP to reduce IR could be through the reduction of adipose tissue, the size of which is generally correlated with inflammatory factors, IR and metabolic dysfunction [52]. Accordingly, GMP supplementation caused a trend toward increased levels of adiponectin, a key anti-inflammatory adipokine. 

In our previous study, we showed that the plasma levels of LPS, a product of gut bacteria, were elevated in response to a high-fat, high-sucrose diet, suggesting a leaky gut barrier [17]. However, GMP supplementation prevented metabolic endotoxemia and its related complications. An important question we wanted to answer in the present work was concerning the influence of GMP on the gut–liver axis under MetS conditions, given their known bidirectional interaction and communication linked to gut barrier permeability, endotoxemia and bile acid metabolism. By targeting the gut first, our findings demonstrate GMP’s ability to significantly reduce OxS (Nrf2), inflammation (TNF-α, COX-2, NF- κB/IκB ratio, AP-1), endotoxemia (TLR4), ER stress (p-PERK, ATF6, IRE1, GRP78) and local IR (p-Akt/Akt, p-p38/p38, p-SAPK/SAPK). In the second step, we were able to observe a similar GMP-mediated improvement in the liver regarding OxS (GPx, Nrf2), inflammation (TNF-α, COX-2, NF-κB/IκB ratio, AP-1), apoptosis (Bcl2, BAX) and local IR (p-p38/p38, p-SAPK/SAPK). It is likely that GMP acts on certain tight junction proteins (e.g., claudin) to limit intestinal barrier permeability, LPS leakage and metabolic endotoxemia, thereby restoring the liver’s ability to fight inflammation, OxS, LPS detoxification and steatosis. 

In addition, GMP showed a great ability to limit ER stress, which has been highly implicated in the pathogenesis of diverse chronic diseases, including MetS [54]. It is well established that cells subjected to a multitude of ER stressors (e.g., environmental pathogens, inflammation and OxS) accumulate misfolded proteins within the ER lumen [55]. This stockpile activates the UPR, which consists of three main branches governed by the biosensors PERK, ATF6 and IRE1. The signalling cascade takes place when GRP78 dissociates from ER transmembrane transcription factors to bind to misfolded proteins, thereby activating all three UPR branches. In the gut, an HFHF diet raised ATF6, IRE1 and GRP78 without altering PERK protein expression levels. This is in line with previous studies showing that specific components of a high-fat diet such as palmitic acid induce ER stress in epithelial cells, which disturbs gut homeostasis and barrier integrity [56,57]. Noteworthily, GMP downregulated HFHF diet-induced ER stress marker protein expression to control levels, suggesting a reduced accumulation of misfolded proteins. The efficacy of GMP in depressing IRE1 is of paramount importance because this transcription factor, through its kinase activity, is capable of activating JNK and IκB, which, in turn, induce AP- 1 and NF-κB, key regulators of the inflammatory response [58]. As the activation of inflammatory signalling pathways blocks insulin action, GMP’s ability to lessen IRE1 probably mediates some of the benefits observed for insulin sensitivity in our study. On the other hand, HFHF feeding-induced obesity and NAFLD are also associated with ER stress in the liver [59]. In the present study, we found that the livers of HFHF diet-fed animals had reduced levels of PERK, IRE1 and GRP78, while no change was noted in GRP94 (the equivalent of GRP78 in the liver) or ATF6 protein expression. GMP supplementation did not result in the modulation of liver ER stress markers, except for phosphorylation levels of PERK. The explanation for this apparent discordance may lie in the evolution of the UPR response, which varies with the nature and intensity of ER stress [60]. Indeed, as in any stress pathway, the initial cellular response aims to promote adaptation to acute ER stress by activating the UPR pathway; however, if the stress becomes chronic, the UPR response will be inhibited and evolve toward apoptosis [61]. In this sense, there is increasing evidence that metabolic disorders such as NAFLD represent conditions causing chronic stress, leading to the failure of the ER stress response [62]. For example, Sasako et al. demonstrated that in obese diabetic mice, there is decreased expression of the chaperones GRP78/BiP and the transcription factor XBP1, normally activated by IRE1 [63]. This failure of the ER stress pathway is associated with a decline in adaptive responses including the activation of chaperones and an increase in proteins involved in apoptosis such as CHOP, caspases, BAX, BAK and Bcl-2 [61,62]. Accordingly, we found increased BAX and reduced Bcl-2 protein levels in HFHF + Bipro mice livers, whereas this was not the case in the gut. Because HFHF + GMP mice had similar protein levels of BAX and Bcl-2 as chow-fed mice, we hypothesize that GMP limited the progression of a failed UPR response, consistent with the improvement in overall liver health observed in GMP-treated mice. To validate these findings and further our understanding of hepatic ER stress during HFHF feeding, future investigations should evaluate the protein expression of additional major transcription factors involved in the UPR response such as ATF4 and XBP1. 

Indeed, GMP improved overall liver health, as evidenced by lower NAFLD scores and TG and TC levels and the modulation of fatty acids profiles. Importantly, GMP animals exhibited lower concentrations of total, saturated, mono-unsaturated, *n*-7 and *n*-9 fatty acids compared to Bipro animals. Accordingly, the activity of SCD1-Δ9 as well as the lipogenesis index were found to be reduced in the GMP group. In the literature, it was reported that SCD1 gene knockout mice were thinner and did not experience as much weight gain as control mice when subjected to a high-fat diet or when leptin-deficient [64]. This effect seems to be mediated by several mechanisms, including decreased lipid synthesis, increased beta oxidation, thermogenesis or insulin sensitivity in the liver [64], which is in agreement with the current and our previous findings [13].

The next question was whether other factors, including gut microbiota, SCFAs and bile acids, intervene upstream in response to GMP to promote gut–liver axis integrity and normalize metabolic derangements. As expected, HFHF feeding resulted in notable changes in the microbiome composition and SCFA production compared to chow-fed mice, but there were no overt differences between the HFHF + GMP and HFHF + Bipro groups. Accordingly, we observed only subtle changes in bile acid metabolism and bile acid metabolism-related genes in response to GMP treatment. Although some studies have highlighted the potential of GMP to improve gut dysbiosis [26,65], others have reported no beneficial effects of GMP on gut microbiota composition or SCFA synthesis [23,66,67,68]. For example, in a mouse model “humanized” with human fecal microbiota, GMP showed no overall advantage over a lactose-free control on microbiota diversity maintenance or prebiotic activity [68]. Similarly, in a cohort of healthy adults, Wernlund et al. showed that 4-week GMP supplementation did not result in significant change in gut microbiota composition in comparison with control skim milk supplementation [67]. We hypothesize that given the fact that GMP is absorbed intact by the host, there may be limited amounts reaching the microbiota in the lower intestine and, thus, the microbiome would be primarily impacted by the excess dietary fat/sugar and not by GMP intervention itself. Nonetheless, we were able to identify several members of the family Lachnospiraceae as showing different enrichment patterns based on the feeding of GMP. Lachnospiraceae is a diverse family that contains members that have been previously associated with either protection or the development of obesity [69,70,71]. This could be due to their varied ability to use different carbon sources and produce downstream effectors such as SCFAs. Indeed, while we noted an overall increase in the amount of 2-methyl butyric acid and isovaleric acid in HFHF diet-fed animals, there were no differences seen between the HFHF + GMP and HFHF + Bipro groups. Lachnospiraceae’s high gene content diversity may have also contributed to our inability to detect differences in the functional pathways inferred from 16S amplicon sequencing data as these were the predominant taxa found to be differentially abundant [71]. More direct measurements of microbial function such as metatranscriptomics or metaproteomics may be required to unravel the impact that the microbiota may be playing during GMP administration. 

Nonetheless, our results suggest that the microbiota may play a secondary role compared to GMP’s direct impacts on host physiology. This could actually improve the potential translatability of GMP into widespread human clinical use. Previous attempts at translating dietary-based interventions that rely on manipulating the microbiome have had mixed success, likely due to the heterogeneity seen between human microbiotas, resulting in unequal microbial responses to any single compound [72,73]. If GMP can exert its beneficial effects in a largely microbiota-independent manner, the potential confounding caused by microbial heterogeneity between patients could be avoided and allow for a wider population to be targeted for treatment.

Few prior studies have documented the ability of GMP to target all components of MetS and, to our knowledge, none have explored its mechanisms of action in the gut–liver axis. Therefore, our research represents a significant contribution to advancing the understanding of the bioactive effects of this peptide on metabolic health. However, our study also encountered certain limitations, notably, the exclusive use of male animals. We opted for male mice primarily because female mice typically exhibit resistance to diet-induced obesity models [74,75]. Although females tend to gain more weight than males on a high-fat diet, they do not typically develop IR or systemic inflammation [75]. Since these metabolic dysregulations are integral to MetS, it was crucial for the animals to manifest these traits to evaluate the potential preventive role of GMP supplementation. Nevertheless, selecting only male subjects for our animal experiment carries several limitations. Given the sexual dimorphism observed in various components of MetS and the increasing evidence of differences in drug responses between males and females, validating our findings in female subjects in subsequent studies will be imperative to ensure their generalizability.

## 5. Conclusions

In summary, our findings underscore the potential of the natural bioactive compound GMP to ameliorate metabolic health in a diet-induced MetS mouse model. Specifically, GMP supplementation led to notable improvements in intestinal and hepatic homeostasis by mitigating inflammation, OxS and ER stress through the regulation of potent transcription factors. While the beneficial effects of GMP do not seem to involve mechanisms driven by the gut microbiota, we hypothesize that the peptide’s actions stem from direct interaction with various receptors within the gut or post-absorption. Although previous studies have suggested that GMP is predominantly found in circulation, recent research has revealed that a significant portion of GMP is degraded into smaller peptides of 11 to 20 amino acids within 1 to 3 h post-consumption [76]. This finding raises the possibility that the peptide’s effects may be primarily mediated by its interaction with various receptors directly within the gut rather than following absorption into circulation. Further investigations are warranted to elucidate the primary mode of action of the peptide.

## Data Availability

All data supporting the findings of this study are available within the paper and its Supplementary Information.

## Supplementary Materials

The following supporting information can be downloaded at https://www.mdpi.com/article/10.3390/nu16060871/s1: Figure S1. Chronological diagram explaining the study design; Figure S2. Glycomacropeptide prevented low-grade inflammation; Figure S3. High-fat, high-fructose diet did not appear to impact species evenness; Figure S4. High-fat, high-fructose diet led to reduced species richness regardless of glycomacropeptide supplementation and distinct microbiotas compared to chow in the cecum; Figure S5. High-fat, high-fructose diet led to increased levels of specific SCFAs regardless of glycomacropeptide supplementation; Table S1. List of primers used for RT-qPCR analysis; Table S2. List of primary antibodies and dilution used for Western blot analysis; Table S3: PERMANOVA results from various mouse groupings; Table S4: Differentially abundant taxa based on single timepoint; Table S5: Differentially abundant taxa over time; Table S6. Hepatic fatty acid profile was profoundly modulated by glycomacropeptide administration.
