# Supplementary material for: Glycomacropeptide as an Efficient Agent to Fight Pathophysiological Mechanisms of Metabolic Syndrome
Source: Nutrients. 2024 Mar 17;16(6):871. doi: 10.3390/nu16060871 (PMC10974946; doi:10.3390/nu16060871)
Supplement: Supplementary file 1 [file nutrients-16-00871-s001.zip › Supplemental_tables S1-S2 & S6.pdf]

TABLE S1. List of primers used for RT-qPCR analysis

	Primers
**ZO-1**	Forward : TGCTGTTCAGCAGCTAAGGA
	Reverse : AGCTCTTGGGTCATGCACTT
**Claudin 1**	Forward : TGGAAGATGATGAGGTGCAG
	Reverse : CCTGGCCAAATTCATACCTG
**Occludin**	Forward : GTTGATCCCCAGGAGGCTAT
	Reverse : GGGGGCTGTTCATCATAAAT
**FXRα**	Forward : GGCCTCTGGGTACCACTACA
	Reverse : ACATCCCCATCTCTTTGCAC
**RXRα**	Forward : CTTTGACAGGGTGCTAACAGAGC
	Reverse : ACGCTTCTAGTGACGCATACACC
**FGF15**	Forward : GCTGGTCCCTATGTCTCCAA
	Reverse : CAGTCCATTTCCTCCCTGAA
**SHP**	Forward : AGCTGGGTCCCAAGGAGTAT
	Reverse : CTTGAGGGTAGAGGCCATGA
**CYP7A1**	Forward : AGGACTTCACTCTACACC
	Reverse : TGGTCTTTGCTTTCCCACTT
**Actin**	Forward: GACAGGATGCAGAAGGAGATTACTG
	Reverse: CCACCGATCCACACAGAGTACTT

TABLE S2. List of primary antibodies and dilution used for Western blot analysis

Antibodies	Molecular weight, antibody dilution and company name
AKT	60 kDa, 1/1000, Cell signaling
Activator protein-1 (AP-1)	110 kDa, 1/1000, ThermoFisher Scientific
Activating transcription factor 6 (ATF6)	90 kDa, 1/1000, Proteintech
β-actin	43 kDa, 1/250,000, Sigma-Aldrich
Bcl2-Associated X Protein (BAX)	21 kDa, 1/1000, Invitrogen
B-cell lymphoma 2 (Bcl-2)	26 kDa, 1/1000, Abcam
Cyclooxygenase-2 (COX-2)	70 kDa, 1/1000, Novus
Glyceraldehyde 3-phosphate dehydrogenase (GAPDH)	34 kDa, 1/1000, Invitrogen
Glucose-regulated protein 78 (GRP78)	72 kDa, 1/1000, Abcam
Glucose-regulated protein 94 (GRP94)	100 kDa, 1/1000, Invitrogen
Inhibitor of kappa B (IκB)	39 kDa, 1/1000, Cell Signaling Biotechnology
Inositol-requiring enzyme 1 (IRE1)	110 kDa, 1/1000, Abcam
Lamin A+C	70/74 kD, 1/1000, Abcam
Nuclear factor kappa-B (NF-κB)	75 kDa, 1/5000, Santa Cruz Biotechnology
Nuclear factor erythroid-2-related factor 2 (NRF2)	75 kDa, 1/1000, Abcam
Phospho-AKT (p-AKT)	60 kDa, 1/1000, ThermoFisher scientific
Protein Kinase RNA-Like ER Kinase (PERK)	140 kDa, 1/1000, Proteintech
Phospho PERK (p-PERK)	130 kDa, 1/500, Invitrogen
Phospho p38 MAP kinase (p-p38)	43 kDa, 1/1000, Cell signaling
p38 MAPK alpha (p38)	43 kDa, 1/1000, ThermoFisher scientific
Phospho Stress-Activated Protein Kinases/Jun amino-terminal Kinases (p-SAPK/JNK)	46 kDa, 1/500, Cell signalling
Stress-activated protein kinases/Jun amino-terminal kinases (SAPK/JNK)	46 kDa, 1/1000, Cell signalling
Toll-like receptor (TLR)-4	65 kDa, 1/1000, Abcam
Tumor necrosis factor-alpha (TNFα)	26 kDa, 1/1000, ThermoFisher scientific

TABLE S6. Hepatic fatty acid profile is profoundly modulated by glycomacropeptide administration

	Chow (*N*=10)			Bipro (*N*=10)			GMP (*N*=10)		
**SFA**	**ug/mg proteins**								
C14:0	11,38	±	1,48	29,90	±	4,39**	18,28	±	2,30
C15:0	3,13	±	0,33	4,93	±	0,55*	3,96	±	0,38
C16:0	637,26	±	62,28	1553,78	±	229,66***	1023,49	±	87,54
C18:0	231,07	±	16,16	314,57	±	23,96**	283,42	±	12,62
C20:0	5,42	±	0,63	13,92	±	1,89***	11,21	±	1,12#
C22:0	13,03	±	0,90	14,39	±	1,07	15,26	±	0,99
C24:0	7,07	±	0,45	3,98	±	0,30***	4,30	±	0,29
**MUFA**									
C16:1n7	78,35	±	13,27	155,41	±	27,00**	93,27	±	12,12
C18:1n7	69,95	±	11,26	178,28	±	31,97***	100,41	±	11,97
C18:1n9	469,11	±	70,79	2062,01	±	362,87***	1220,13	±	132,57
C20:1n9	12,63	±	1,79	50,17	±	9,47***	27,65	±	3,54
C24:1n9	8,77	±	0,86	24,46	±	3,80***	16,41	±	1,63
**PUFA**									
C18:3n3	26,04	±	3,64	21,39	±	2,30	17,91	±	1,59
C20:3n3	1,01	±	0,12	1,80	±	0,21	1,43	±	0,15
C20:5n3	7,92	±	1,08	8,44	±	0,87	7,41	±	0,55
C22:5n3	13,95	±	1,65	20,44	±	2,65	15,74	±	1,36
C22:6n3	121,02	±	10,45	151,53	±	14,94	135,82	±	6,03
C18:2n6	618,43	±	72,13	870,26	±	101,90	693,30	±	51,55
C18:3n6	7,20	±	0,74	15,98	±	2,12***	12,73	±	1,43#
C20:2n6	8,87	±	0,85	18,05	±	2,35***	12,20	±	1,10
C20:3n6	30,05	±	3,71	50,71	±	6,51**	35,13	±	3,53
C20:4n6	229,48	±	16,18	305,34	±	24,33*	267,73	±	16,56
C22:4n6	8,32	±	0,40	6,58	±	0,47*	6,51	±	0,39#
**Total FAs**	2632,88	±	282,75	5901,38	±	838,67***	4042,20	±	329,05#
**Total SFA**	916,24	±	80,83	1947,42	±	257,20***	1369,40	±	99,44#
**Total MUFA**	641,88	±	97,72	2481,64	±	435,11****	1550,19	±	178,59#
**Total PUFA**	1074,75	±	109,19	1472,32	±	156,07	1207,40	±	76,97
**Total n-3**	171,25	±	16,38	204,17	±	20,50	178,71	±	8,48
**Total n-6**	903,44	±	92,93	1267,80	±	136,03*	1028,50	±	69,02
**Total n-7**	148,30	±	24,50	333,69	±	58,65**	193,68	±	24,03#
**Total n-9**	490,58	±	73,41	2136,98	±	375,84***	1264,38	±	137,40#

	Chow (*N*=10)			Bipro (*N*=10)			GMP (*N*=10)		
**RATIOS**									
PUFA/saturated	1,16	±	0,03	0,79	±	0,04*	0,89	±	0,02
16:1n-7/18:2n-6	0,12	±	0,01	0,17	±	0,01****	0,13	±	0,01#
FADS1-Δ5 (20:4n-6/20:3n-6)	8,24	±	0,62	6,53	±	0,47	8,32	±	0,90
FADS2-Δ6 (18:3n-6/18:2n-6)	0,01	±	0,00	0,02	±	0,00****	0,02	±	0,00
SCD1-Δ7 (16:1n-7/16:0)	0,12	±	0,01	0,10	±	0,00	0,09	±	0,01
SCD1-Δ9 (18:1n-9/18:0)	1,95	±	0,21	6,25	±	0,84****	4,29	±	0,39#
ELOV3 (C24:1n-9/C18:1n-9)	0,02	±	0,00	0,01	±	0,00****	0,01	±	0,00
ELOV5 (C20:5n-3/C18:3n-3)	0,30	±	0,01	0,40	±	0,01****	0,43	±	0,03
ELOV5 (C20:4n-6/C18:2n-6)	0,39	±	0,02	0,37	±	0,02	0,39	±	0,02
ELOV6 (C18:0/C16:0)	0,37	±	0,02	0,23	±	0,02***	0,29	±	0,03
Lipogenesis index (C16:0/C18:2n6)	1,06	±	0,03	1,73	±	0,09****	1,47	±	0,05#

Abbreviations: ELOV, elongation of very long chain fatty acids; FA, fatty acids; FADS, fatty acid desaturase; MUFA, monounsaturated fatty acids; PUFA, polyunsaturated fatty acids; SCD, stearoyl-CoA Desaturase; SFA, saturated fatty acids

Free fatty acid determination was performed in liver tissue of chow and HFHF-fed mice supplemented with either Bipro or GMP. Results are expressed as concentration of fatty acid (ug/mg proteins) determined using a fatty acid standard curve (n = 10/group). Results represent the mean ± SEM. One-way analysis of variance was performed followed by Tukey’s multiple comparisons test. Versus chow: * P <0.05, ** P <0.01, *** P <0.001, **** P <0.0001, versus HFHF+Bipro # P <0.05
