# Supplementary material for: Glycomacropeptide as an Efficient Agent to Fight Pathophysiological Mechanisms of Metabolic Syndrome
Source: Nutrients. 2024 Mar 17;16(6):871. doi: 10.3390/nu16060871 (PMC10974946; doi:10.3390/nu16060871)
Supplement: Supplementary file 1 [file nutrients-16-00871-s001.zip › Supplementary data (Figures S1-S5).pdf]

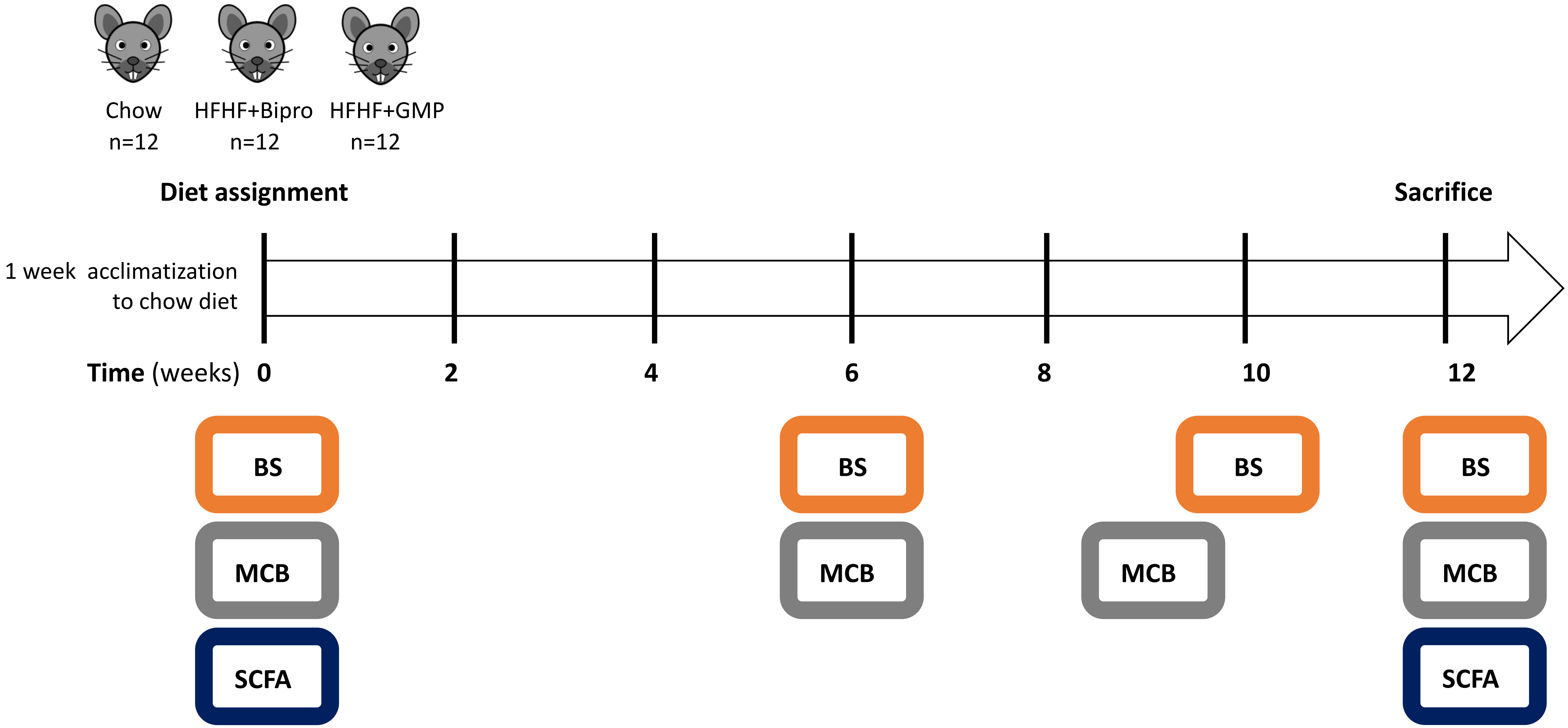
Chow
n=12
HFHF+Bipro
n=12
HFHF+GMP
n=12
Diet assignment
Sacrifice
1 week acclimatization
to chow diet
Time (weeks)
0
2
4
6
8
10
12
BS
MCB
SCFA
BS
MCB
MCB
BS
BS
MCB
SCFA

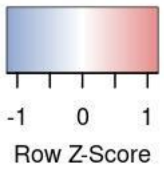

	Chow	Bipro	GMP
Eotaxin	1612.16	1804.48	1864.62
G.CSF	527.96	836.22	488.77
IL.1.	49.45	113.35	38.39
IL.1..1	5.38	29.62	6.27
IL.2	29.41	42.21	11.26
IL.4	0.26	1.26	0.35
IL.5	5.61	6.53	7.64
IL.6	5.46	10.78	4.48
IL.7	6.01	14.99	2.79
IL.9	18.85	18.13	15.42
IL.10	4.20	11.99	5.38
IL.12.p4	12.50	35.47	10.12
IL.12.p7	9.87	17.91	135.93
IL.13	46.85	79.52	50.60
IL.15	70.52	223.03	40.67
IL.17	16.32	13.72	4.59
IP.10	71.63	80.24	63.89
KC	252.78	478.74	265.20
LIF	0.69	4.24	0.57
LIX	369.99	795.58	268.18
MCP.1	35.08	93.68	27.52
M.CSF	7.89	20.31	6.61
MIG	219.03	297.32	206.50
MIP1..	122.16	138.23	67.54
MIP1...1	29.19	48.46	21.75
MIP.2	89.06	145.28	51.19
RANTES	27.16	37.64	30.56
TNF.	8.06	22.12	7.64
VEGF	1.09	1.54	0.63

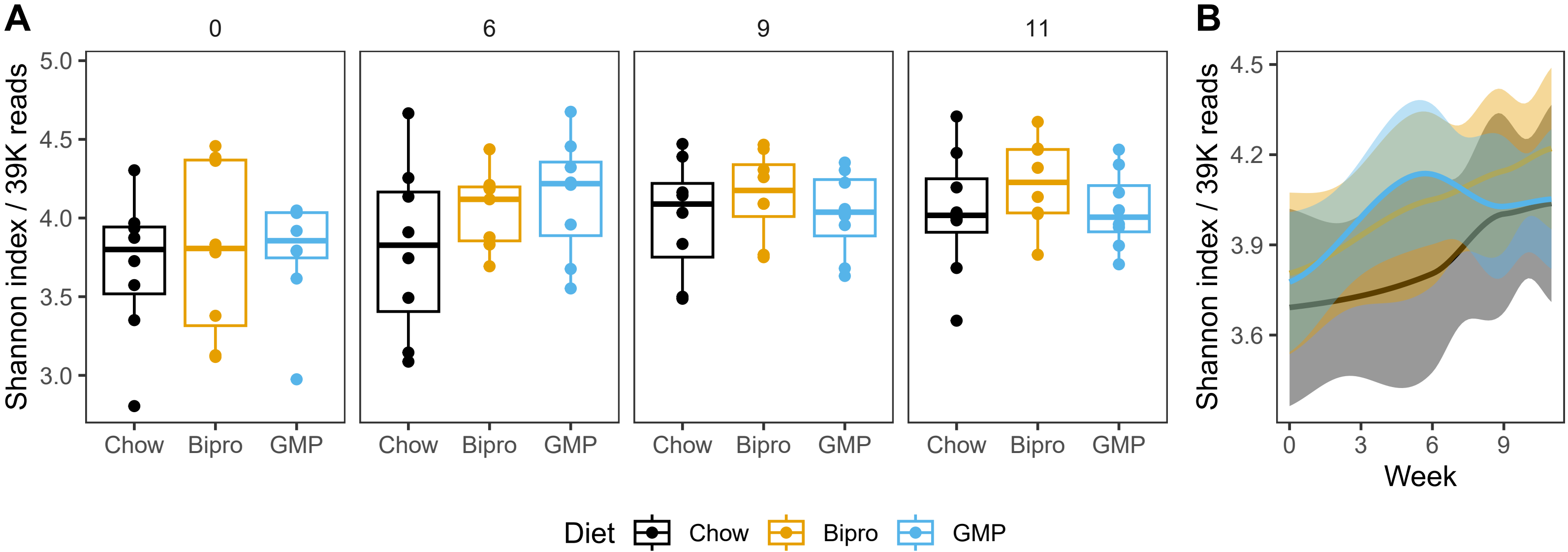
A
0
6
9
11
Shannon index / 39K reads
5.0
4.5
4.0
3.5
3.0
Chow
Bipro
GMP
B
4.5
4.2
3.9
3.6
0
3
6
9
Week
Diet
Chow
Bipro
GMP

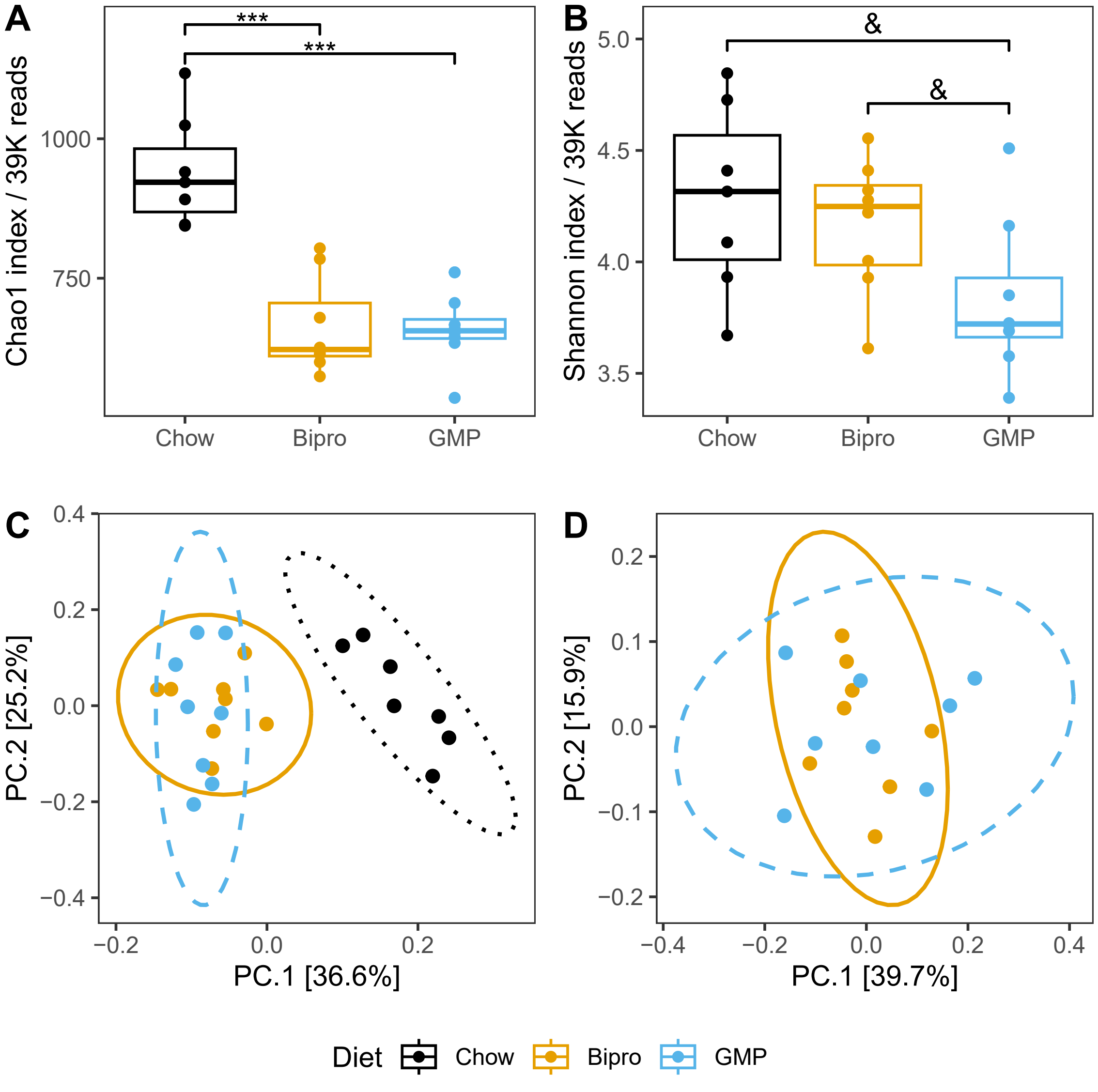
A
Chao1 index / 39K reads
***
***
1000
750
Chow
Bipro
GMP
B
Shannon index / 39K reads
&
&
5.0
4.5
4.0
3.5
Chow
Bipro
GMP
C
PC.2 [25.2%]
0.4
0.2
0.0
−0.2
−0.4
−0.2
0.0
0.2
PC.1 [36.6%]
D
PC.2 [15.9%]
0.2
0.1
0.0
−0.1
−0.2
−0.4
−0.2
0.0
0.2
0.4
PC.1 [39.7%]
Diet
Chow
Bipro
GMP

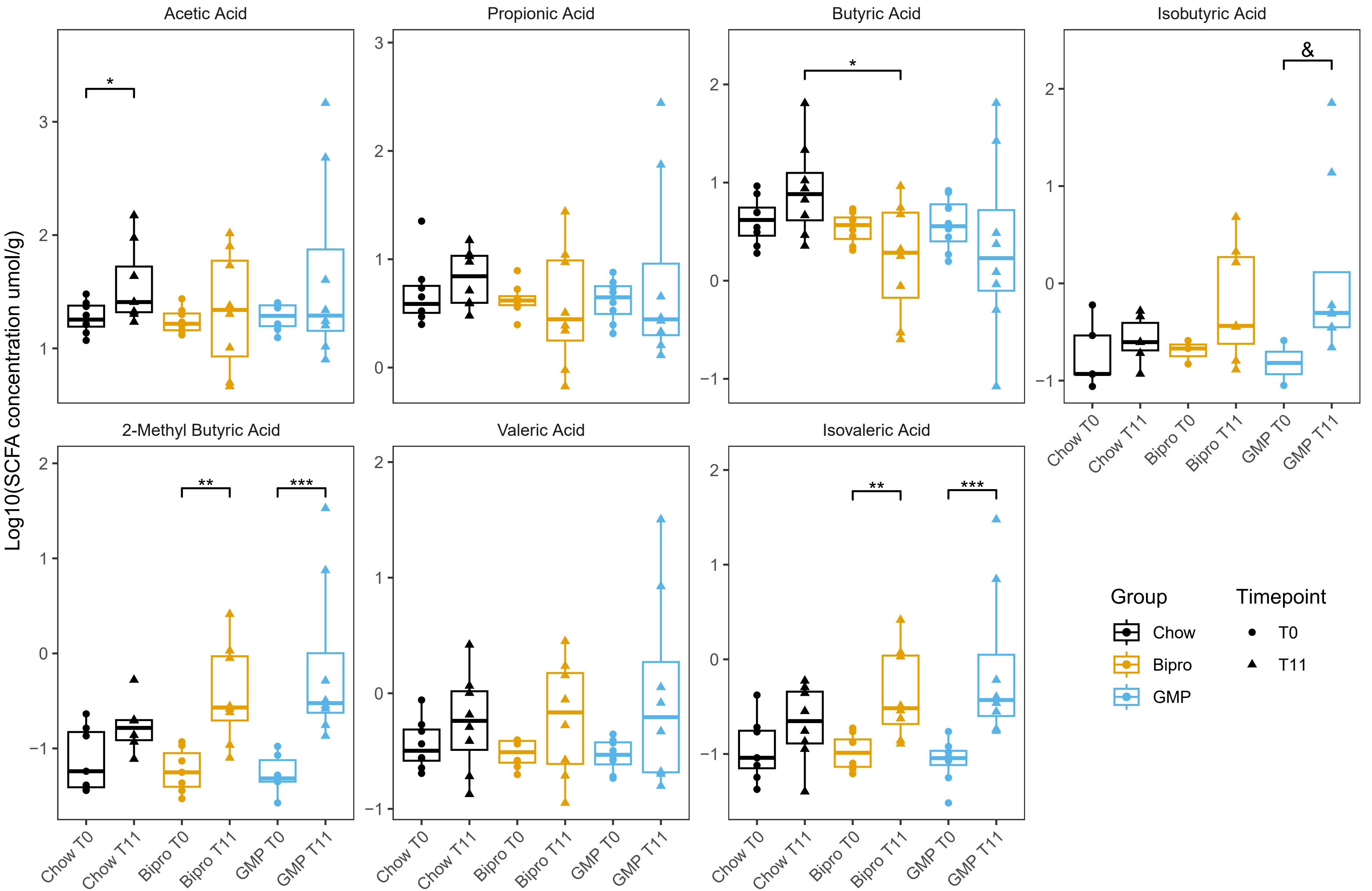
Acetic Acid
Propionic Acid
Butyric Acid
Isobutyric Acid
2-Methyl Butyric Acid
Valeric Acid
Isovaleric Acid
Log10(SCFA concentration umol/g)
Chow T0
Chow T11
Bipro T0
Bipro T11
GMP T0
GMP T11
*
**
***
&
Group
Chow
Bipro
GMP
Timepoint
T0
T11

SUPPLEMENTARY FIGURE LEGENDS

Figure S1: Chronological diagram explaining the study design
After statistical analysis of data and exclusion of outlyers animals, 8 animals per group were kept for further analyses.
HFHF: high-fat high fructose diet; BS: blood sampling; MCB: microbiota sampling; SCFA: short chain fatty acid sampling

Figure S2: Glycomacropeptide prevents low-grade inflammation
Bio-Plex quantification of cytokines and chemokines in plasma of chow and HFHF-fed mice supplemented with either Bipro or GMP. Data are represented as a heatmap with clustering with median concentrations in pg/mL (inside the boxes) determined using a synthetic cytokine standard curve (n = 8/group).

Figure S3. High fat high fructose diet does not appear to impact species evenness
Shannon index between feeding groups at each sampling timepoint (A) and over time (B). Boxplots (A) show the interquartile range with the median represented as a line. Changes over time (B) are represented as LOWESS fits to the data with shaded regions denoting 95% confidence intervals. $P<0.5$*, $P<0.01$**, $P<0.001$*** (note: only significant associations are plotted).

Figure S4. High fat high fructose diet leads to reduced species richness regardless of glycomacropeptide supplementation and distinct microbiotas as compared to Chow in the cecum
Chao1 index (A), Shannon index (B) and weighted Unifrac principal coordinate analysis (C,D) for cecum samples. Boxplots show the interquartile range with the median represented as a line. Ellipses represent the 95% confidence intervals for each group. $P <$ 0.1&, < 0.5*, 0.01** and 0.001*** (note: only significant associations are plotted).

Figure S5. High fat high fructose diet leads to increased levels of specific SCFAs regardless of glycomacropeptide supplementation
Levels of measured SCFAs in each feeding group at T0 and after 11 weeks of the dietary intervention. Boxplots show the interquartile range with the median represented as a line. $P <$ 0.1&, < 0.5*, 0.01** and 0.001*** (note: only significant associations are plotted).
